# AI demonstrates comparable diagnostic performance to radiologists in MRI detection of anterior cruciate ligament tears: a systematic review and meta-analysis

**DOI:** 10.1007/s00330-025-12020-2

**Published:** 2025-09-25

**Authors:** Saran Singh Gill, Taha Haq, Yi Zhao, Mihailo Ristic, Dimitri Amiras, Chinmay Madhukar Gupte

**Affiliations:** 1 Imperial College London, London, United Kingdom; 2 Imperial College Healthcare NHS Trust, London, United Kingdom

**Keywords:** AI, Artificial intelligence, ACL, Anterior cruciate ligament, Diagnosis

## Abstract

**Introduction:**

Anterior cruciate ligament (ACL) injuries are among the most common knee injuries, affecting 1 in 3500 people annually. With rising rates of ACL tears, particularly in children, timely diagnosis is critical. This study evaluates artificial intelligence (AI) effectiveness in diagnosing and classifying ACL tears on MRI through a systematic review and meta-analysis, comparing AI performance with clinicians and assessing radiomic and non-radiomic models.

**Methods:**

Major databases were searched for AI models diagnosing ACL tears via MRIs. 36 studies, representing 52 models, were included. Accuracy, sensitivity, and specificity metrics were extracted. Pooled estimates were calculated using a random-effects model. Subgroup analyses compared MRI sequences, ground truths, AI versus clinician performance, and radiomic versus non-radiomic models. This study was conducted in line with Preferred Reporting Items for Systematic Reviews and Meta-Analyses (PRISMA) protocols.

**Results:**

AI demonstrated strong diagnostic performance, with pooled accuracy, sensitivity, and specificity of 87.37%, 90.73%, and 91.34%, respectively. Classification models achieved pooled metrics of 90.46%, 88.68%, and 94.08%. Radiomic models outperformed non-radiomic models, and AI demonstrated comparable performance to clinicians in key metrics. Three-dimensional (3D) proton density fat suppression (PDFS) sequences with < 2 mm slice depth yielded the most promising results, despite small sample sizes, favouring arthroscopic benchmarks. Despite high heterogeneity (I² > 90%).

**Conclusion:**

AI models demonstrate diagnostic performance comparable to clinicians and may serve as valuable adjuncts in ACL tear detection, pending prospective validation. However, substantial heterogeneity and limited interpretability remain key challenges. Further research and standardised evaluation frameworks are needed to support clinical integration.

**Key Points:**

***Question*** *Is AI effective and accurate in diagnosing and classifying anterior cruciate ligament (ACL) tears on MRI?*

***Findings*** *AI demonstrated high accuracy (87.37%), sensitivity (90.73%), and specificity (91.34%) in ACL tear diagnosis, matching or surpassing clinicians. Radiomic models outperformed non-radiomic approaches.*

***Clinical relevance*** *AI can enhance the accuracy of ACL tear diagnosis, reducing misdiagnoses and supporting clinicians, especially in resource-limited settings. Its integration into clinical workflows may streamline MRI interpretation, reduce diagnostic delays, and improve patient outcomes by optimising management.*

## Background

The anterior cruciate ligament (ACL) is the second most commonly injured knee ligament, accounting for up to half of all knee injuries presenting with acute swelling. It affects 1 in 3500 people annually [1]. The incidence of ACL tears is rising, particularly in children and adolescents, with paediatric ACL reconstructions increasing by over 40% in Norway between 2005 and 2021 [2, 3]. Magnetic Resonance Imaging (MRI) remains the gold standard for ACL tear diagnosis, achieving high detection rates (95.85%) and accuracy (94.87%) [4, 5]. Although complex injury presentations and partial tears can complicate interpretation, contributing to interobserver variability and misclassification [4, 6]. Furthermore, the rates of missed diagnosis and misdiagnosis have been reported to be 13% and 10%, respectively [7]. If left untreated, there is a significantly increased risk of secondary injury to surrounding structures, including the menisci, as well as chronic knee instability and greater risk of osteoarthritis [8, 9].

ACL tears are typically evaluated using MRI sequences such as proton density fat-saturated (PDFS), T2-weighted, and T1-weighted imaging, with PDFS offering superior soft tissue contrast and fluid sensitivity, making it particularly effective for visualising ligamentous structures [10]. Both 2D and 3D imaging protocols are employed; while 3D turbo spin-echo (TSE) sequences provide isotropic resolution and allow for multiplanar reconstructions, routine 2D TSE remains the standard in most clinical settings [11, 12]. Notably, diagnostic performance for ACL tears is comparable between 2D and 3D TSEs, with reported sensitivities of 93% and specificities of 80–85% [13]. Despite advances in imaging, diagnostic accuracy can vary substantially depending on radiologist experience, with junior readers typically demonstrating lower sensitivity, specificity, and longer reporting times compared to more experienced colleagues [14, 15].

The use of AI, specifically deep learning, to aid the diagnosis of an ACL tear on MRIs has been gaining traction, aiming to enhance the outcomes of radiologists in clinical practice, particularly in centres with less experience interpreting MRI scans [16–19]. Radiomics has emerged as a notable area of interest within AI-based detection models using clinical imaging, particularly for musculoskeletal conditions such as ACL tears [20, 21]. Although not strictly defined, radiomics refers to the extraction of quantitative, reproducible features from diagnostic images, capturing complex patterns and textures that may be imperceptible to the human eye [22]. By leveraging radiomic features, models can interpret MRIs with greater accuracy, detect subtle abnormalities, and define more accurate regions of interest (ROIs) for diagnosing conditions like ACL tears [23]. Unlike earlier deep learning approaches, radiomics-based models often use more focused datasets with carefully delineated ROIs, allowing for finer-grained analysis [24–28]. While this methodological specificity may introduce some bias, given that such detailed image annotation is not routine in everyday clinical workflows, it represents an important step toward improving diagnostic precision. As such, radiomic-based approaches remain a promising avenue for further investigation, particularly in efforts to bridge the gap between algorithmic performance and clinical utility.

While prior systematic reviews have evaluated AI performance in diagnosing and classifying ACL tears on MRI, many are limited by small sample sizes, exclusion of relevant models, and insufficient analysis of key methodological issues such as model interpretability and the quality of ground truth (GT) labelling [29–31]. In contrast, our review provides a more recent, comprehensive, and focused synthesis, incorporating evaluation of AI’s performance and potential usage as an adjunct to clinicians. Our study aims to provide the most recent, specific, and comprehensive review of the literature, encompassing various AI approaches, critically examine algorithmic concerns and limitations, and present a quantitative synthesis through meta-analysis.

## Materials and methods

### Search methodology

**Fig. 1:**
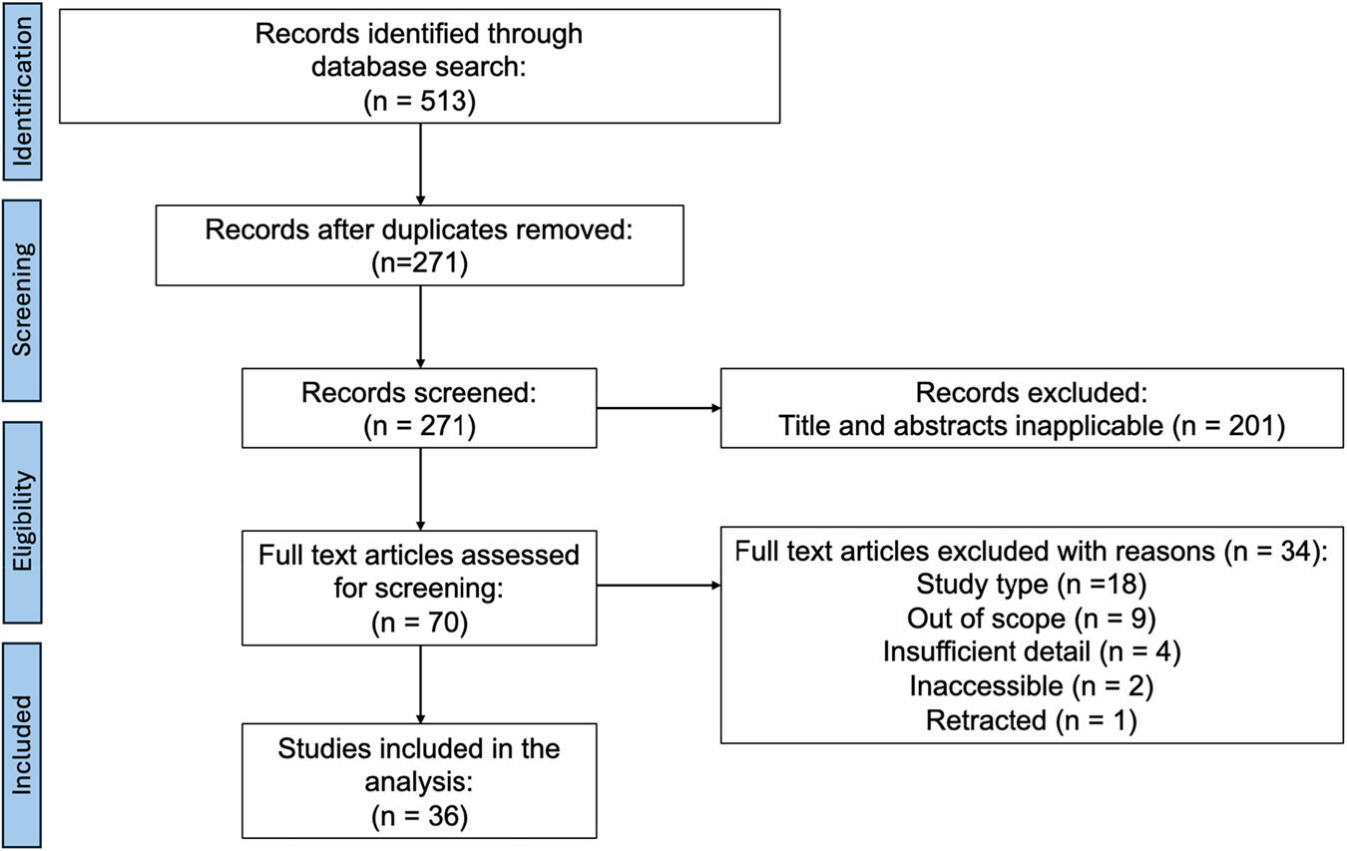
PRISMA. PRISMA diagram outlining the screening process

This systematic review and meta-analysis adhered to the PRISMA-P 2015 checklist and was registered on International Prospective Register of Systematic Reviews (PROSPERO) (CRD42024593390) [32]. A comprehensive literature search was conducted from database inception to September 22, 2024, across major databases using medical subject headings (MeSH) terms and Boolean operators, without applying language or publication filters. However, only studies published in English were included during screening (Fig. 1). The search strategy, developed with an institutional librarian, addressed the question: “Is AI effective and accurate in diagnosing and classifying ACL tears on MRI?” (Table s1).

### Study selection and inclusion criteria

Articles were uploaded into COVIDENCE for screening by two reviewers, with discrepancies resolved by a third [33]. Reference lists were manually searched. Titles and abstracts were assessed using predefined inclusion and exclusion criteria (Tables s2, s3), with full-text screening for AI-based MRI ACL diagnosis studies.

### Data extraction

Two reviewers independently extracted data, including sensitivity, specificity, accuracy, precision, F1, and AUC (Table s4). Missing or unclear data prompted author correspondence. For the purpose of this review, diagnosis was defined as the binary identification of ACL tear presence, distinguishing between torn and intact ligaments. Classification referred to the categorisation of ACL status into more detailed subgroups, such as intact, partially torn, or fully torn, based on the level of structural disruption.

### Critical appraisal

Methodological rigour was assessed using the CLAIM-AI checklist, while the Prediction model Risk Of Bias ASsessment Tool (PROBAST) framework evaluated Risk of Bias (RoB) [34, 35]. Two evaluators performed assessments, with discrepancies resolved by a third. Applicability and data reliability were reviewed.

### Meta-analysis

Pooled estimates for AI diagnostic accuracy, specificity, and sensitivity were computed, with 95% CIs calculated when unavailable, using the Wald method based on the binomial standard error formula. A random-effects model accounted for heterogeneity (I²) [36]. Validation data were extracted where possible. Subgroup analyses compared clinician performance to AI models, classification of ACL tears, radiomic models, MRI types, and GTs, requiring a minimum of three studies. Classification-based models, analysed as a subgroup, were defined as diagnosing full-thickness, partial, or no ACL tear. Standardised mean differences (SMDs) were used to compare AI and clinician performance metrics, calculated as the difference in means divided by the pooled standard deviation (Cohen’s d). When unavailable, 95% confidence intervals were estimated using the SMD variance. Subgroup analyses were conducted only for groups with ≥ 3 studies. Results were visualised via forest plots, with statistical significance set at *p* < 0.05. Analyses were conducted in RStudio (version 2024.04.2 + 764; R version 4.4.0), using the ‘meta’ (version 8.1-0) and ‘metafor’ (Version 4.6-0) packages.

### Publication bias

Publication bias was assessed in the overall specificity, sensitivity and accuracy using Egger’s tests and visualised using Funnel plots using the ‘meta (version 8.1-0)’ package.

## Results

Out of 271 studies screened, 36 studies (52 models) were included in the review and meta-analysis [18, 24–28, 37–66]. Study characteristics and performance metrics are summarised in Tables 1, s5–s7. High heterogeneity was observed across forest plots. Of these studies, 24/36 (66.67%) were fully automated. The remainder included 11/36 (30.56%) that required manual pre-processing, 1/36 (2.78%) semi-automatic, and 1/36 (2.78%) supervised automated. A total of 10/36 (27.78%) studies used explainability methods (Table s8). The majority underwent internal validation (28/36, 77.78%), while the remaining 8/36 (22.22%) underwent external validation, 3/8 (37/5%) of which were multinational.

**Fig. 2:**
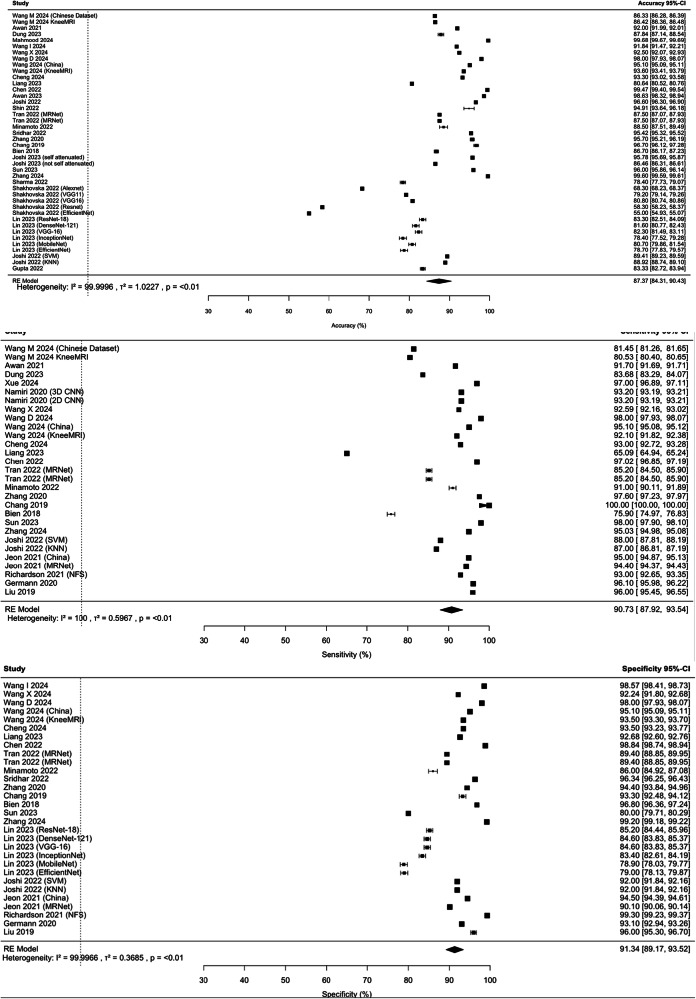
Forest plot of the performance of AI in the diagnosis of ACL tears across studies. The forest plots pertain to the percentage accuracy, sensitivity and specificity

**Fig. 3:**
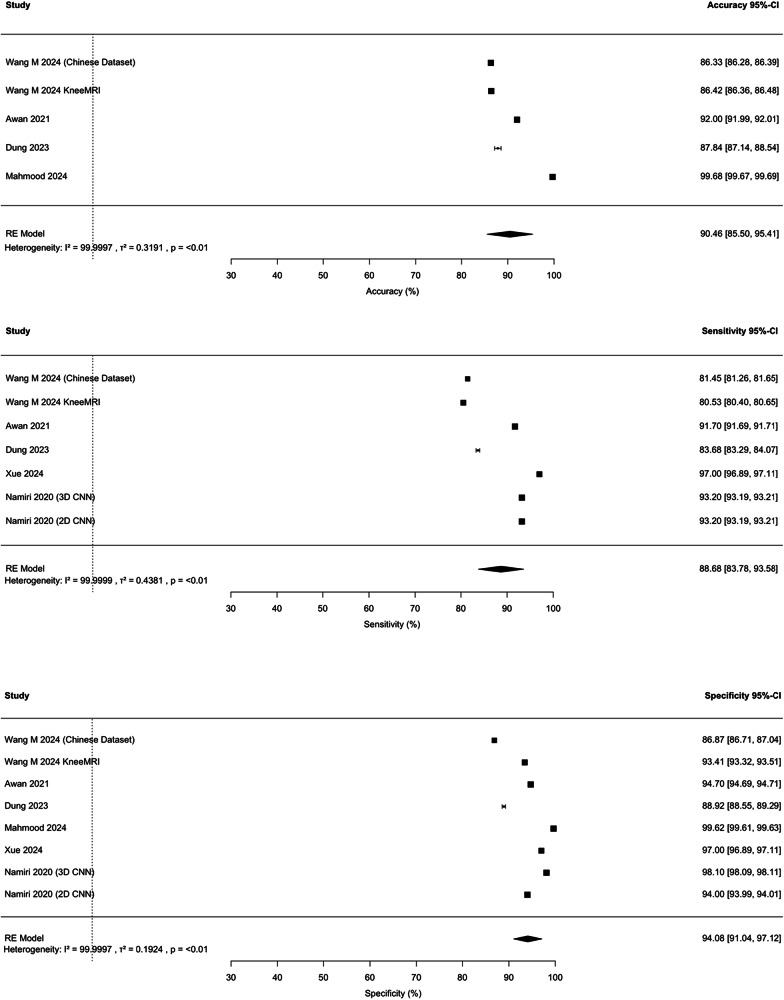
Forest plot of outcomes of AI’s performance in ACL tear classification across studies. The forest plots pertain to the percentage accuracy, sensitivity and specificity

Convolutional Neural Networks (CNNs) were the predominant baseline architecture, utilised in 84.6% (44/52) of cases, while the remaining models used various methodologies, including the Improved Honey Badger Algorithm (IHBA) as a CNN optimiser and Support Vector Machines (SVMs) (Table 1). A critical appraisal of included studies is summarised in Figs. s2 and s3. Funnel plots can be found in Fig. s4. All forest plots can be found in Figs. 2, 3, s5–s22.

**Table 1:** Study characteristics

Study	Title	Country	Type of AI	Domain	Ground truth	*N*	Radiomics model used	Accuracy (%)	Sensitivity (%)	Specificity (%)	Other Metrics
Wang [62] (Chinese Dataset)	One-stop detection of anterior cruciate ligament injuries on magnetic resonance imaging using deep learning with multicenter validation.	China	CNN	Classification	Radiologists	917	No	86.33	81.45	86.87	-
Wang [62] (KneeMRI)	One-stop detection of anterior cruciate ligament injuries on magnetic resonance imaging using deep learning with multicenter validation.	China	CNN	Classification	Radiologists	920	No	86.42	80.53	93.41	-
Awan [42]	Efficient detection of knee anterior cruciate ligament from magnetic resonance imaging using deep learning approach.	Malaysia	CNN	Classification	Orthopaedic surgeons	917	No	92.00	91.70	94.70	Precision: 91.7
Dung [26]	End-to-end deep learning model for segmentation and severity staging of anterior cruciate ligament injuries from MRI.	Vietnam	CNN	Classification	Arthroscopy	50	Yes	87.84	83.68	88.92	-
Mahmood [64]	Acute knee injury detection with magnetic resonance imaging (MRI)	Saudia Arabia	CNN	Classification	KneeMRI Dataset	917	No	99.68	-	99.62	Precision: 99.52
Chen [27]	A transfer learning approach for staging diagnosis of anterior cruciate ligament injury on a new modified MR dual precision positioning of thin-slice oblique sagittal FS-PDWI sequence.	China	TL	Classification	Arthroscopy	664	Yes	-	-	-	Precision: 95.2
Xue [28]	Approaching expert-level accuracy for differentiating ACL tear types on MRI with deep learning.	China	CNN	Classification	Arthroscopy	90	Yes	-	97.00	97.00	-
Namiri [65] (3D CNN)	Deep learning for hierarchical severity staging of anterior cruciate ligament injuries from MRI.	US	DL	Classification	Radiologists	1243	No	-	93.20	98.10	-
Namiri [65] (2D CNN)	Deep learning for hierarchical severity staging of anterior cruciate ligament injuries from MRI.	US	CNN	Classification	Radiologists	1243	No	-	93.20	94.00	-
Wang [37]	Lightweight attentive graph neural network with conditional random field for diagnosis of anterior cruciate ligament tear.	China	CNN	Diagnosis	Orthopaedic surgeons	147	No	91.84	-	98.57	-
Wang [38]	Deep learning-assisted automatic diagnosis of anterior cruciate ligament tear in knee magnetic resonance images.	China	CNN	Diagnosis	Radiologists and orthopaedic surgeons	120	No	92.50	92.59	92.24	AUC: 0.9747
Wang [39]	Improving inceptionV4 model based on fractional-order snow leopard optimization algorithm for diagnosing of ACL tears.	China	FO-LOA	Diagnosis	Arthroscopy	411	No	98.00	98.00	98.00	F1: 98.0
Wang [40] (China)	A deep learning model enhances cliniicians’ diagnostic accuracy to more than 96% for anterior cruciate ligament ruptures on magnetic resonance imaging.	China	CNN	Diagnosis	Arthroscopy	4086	No	95.10	95.10	95.10	AUC: 0.987
Wang [40] (KneeMRI)	A deep learning model enhances clinicians’ diagnostic accuracy to more than 96% for anterior cruciate ligament ruptures on magnetic resonance imaging.	China	CNN	Diagnosis	KneeMRI Dataset	110	No	93.60	92.10	93.50	-
Cheng [25]	Application of machine learning-based multi-sequence MRI radiomics in diagnosing anterior cruciate ligament tears.	China	SVM	Diagnosis	Arthroscopy	178	Yes	93.30	93.00	93.50	AUC: 0.973
Liang [24]	Effective automatic detection of anterior cruciate ligament injury using convolutional neural network with two attention mechanism modules.	China	CNN	Diagnosis	Orthopaedic surgeons	630	No	80.64	65.09	92.68	AUC: 0.8886; F1: 84.26; Precision: 77.41
Chen [41]	Artificial intelligence-assisted diagnosis of anterior cruciate ligament tears from magnetic resonance images: algorithm development and validation study.	Taiwan	CNN	Diagnosis	Radiologist and orthopaedic surgeons	200	No	99.47	97.02	98.84	F1: 96.74; Precision: 96.47
Awan [42]	MGACA-Net: a novel deep learning based multi-scale guided attention and context aggregation for localization of knee anterior cruciate ligament tears region in MRI images.	Malaysia	CNN	Diagnosis	Radiologist	3817	No	98.63	-	-	F1: 97.86; Precision: 98.21
Shin [44]	Development of convolutional neural network model for diagnosing tear of anterior cruciate ligament using only one knee magnetic resonance image.	Republic of Korea	CNN	Diagnosis	Radiologist	34	No	94.91	-	-	AUC: 0.941
Tran [45] (MRNet)	Deep learning to detect anterior cruciate ligament tear on knee MRI: multi-continental external validation.	France	CNN	Diagnosis	Radiologic reports	183	No	87.50	85.20	89.40	AUC: 0.962
Tran [45] (MRNet)	Deep learning to detect anterior cruciate ligament tear on knee MRI: multi-continental external validation.	France	CNN	Diagnosis	Radiologic reports	183	No	87.50	85.20	89.40	AUC: 0.922
Minamoto [46]	Automated detection of anterior cruciate ligament tears using a deep convolutional neural network.	Japan	DCNN	Diagnosis	Arthroscopy	200	No	88.50	91.00	86.00	AUC: 0.942
Sridhar [18]	A torn ACL mapping in knee MRI images using deep convolution neural network with Inception-v3.	India	DCNN	Diagnosis	KneeMRI Dataset	411	No	95.42	-	96.34	Precision: 95.02
Zhang [47]	Deep learning approach for anterior cruciate ligament lesion detection: evaluation of diagnostic performance using arthroscopy as the reference standard.	China	CNN	Diagnosis	Arthroscopy	81	No	95.70	97.60	94.40	AUC: 0.957
Chang [48]	Deep learning for detection of complete anterior cruciate ligament tear.	US	CNN	Diagnosis	Radiologist	60	No	96.70	100.00	93.30	-
Bien [49]	Deep-learning-assisted diagnosis for knee magnetic resonance imaging: development and retrospective validation of MRNet.	US	CNN	Diagnosis	Radiologists	133	No	86.70	75.90	96.80	-
Joshi [43] (self-attenuated)	Anterior cruciate ligament tear detection based on convolutional neural network and generative adversarial neural network	India	CNN	Diagnosis	MRNet Dataset	450	No	95.78	-	-	F1: 94.0; Precision: 95.0
Joshi [43] (not self-attenuated)	Anterior cruciate ligament tear detection based on convolutional neural network and generative adversarial neural network	India	CNN	Diagnosis	MRNet Dataset	450	No	86.46	-	-	F1: 84.0; Precision: 81.0
Sun [51]	Anterior cruciate ligament tear detection based on deep belief networks and improved honey badger algorithm	China	IHBA optimiser on a CNN	Diagnosis	Radiologists	275	No	96.00	98.00	80.00	-
Zhang [47]	A new optimization method for accurate anterior cruciate ligament tear diagnosis using convolutional neural network and modified golden search algorithm	Republic of Korea	CNN	Diagnosis	Knee MRI Dataset	917	No	99.60	95.03	99.20	F1: 94.35; Precision: 98.73
Sharma [53]	A ResNet50-based approach to detect multiple types of knee tears using MRIs	India	CNN	Diagnosis	MRNet Dataset	120	No	78.40	-	-	AUC: 0.842
Shakhovska [54] (Alexnet)	Comparative analysis of backbone networks for deep knee MRI classification models	Ukraine	CNN	Diagnosis	KneeMRI Dataset	1370	No	68.30	-	-	AUC: 0.729; F1: 48.6
Shakhovska [54] (VGG11)	Comparative analysis of backbone networks for deep knee MRI classification models	Ukraine	CNN	Diagnosis	KneeMRI Dataset	1370	No	79.20	-	-	AUC: 0.862; F1: 71.3
Shakhovska [54] (VGG16)	Comparative analysis of backbone networks for deep knee MRI classification models	Ukraine	CNN	Diagnosis	KneeMRI Dataset	1370	No	80.80	-	-	AUC: 0.887; F1: 75.3
Shakhovska [54] (Resnet)	Comparative analysis of backbone networks for deep knee MRI classification models	Ukraine	CNN	Diagnosis	KneeMRI Dataset	1370	No	58.30	-	-	AUC: 0.642; F1: 16.7
Shakhovska [54] (EfficientNet)	Comparative analysis of backbone networks for deep knee MRI classification models	Ukraine	CNN	Diagnosis	KneeMRI Dataset	1370	No	55.00	-	-	AUC: 0.603
Lin [55] (ResNet-18)	A channel correction and spatial attention framework for anterior cruciate ligament tear with ordinal loss	China	CNN	Diagnosis	KneeMRI Dataset	92	No	83.30	-	85.20	F1: 49.8; Precision: 48.8
Lin [55] (DenseNet-121)	A channel correction and spatial attention framework for anterior cruciate ligament tear with ordinal loss	China	CNN	Diagnosis	KneeMRI Dataset	92	No	81.60	-	84.60	F1: 48.6; Precision: 47.1
Lin [55] (VGG-16)	A channel correction and spatial attention framework for anterior cruciate ligament tear with ordinal loss	China	CNN	Diagnosis	KneeMRI Dataset	92	No	82.30	-	84.60	F1: 48.8; Precision: 47.7
Lin [55] (InceptionNet)	A channel correction and spatial attention framework for anterior cruciate ligament tear with ordinal loss	China	CNN	Diagnosis	KneeMRI Dataset	92	No	78.40	-	83.40	F1: 47.1; Precision: 45.4
Lin [55] (MobileNet)	A channel correction and spatial attention framework for anterior cruciate ligament tear with ordinal loss	China	CNN	Diagnosis	KneeMRI Dataset	92	No	80.70	-	78.90	F1: 45.0; Precision: 56.5
Lin [55] (EfficientNet)	A channel correction and spatial attention framework for anterior cruciate ligament tear with ordinal loss	China	CNN	Diagnosis	KneeMRI Dataset	92	No	78.70	-	79.00	F1: 42.8; Precision: 41.5
Joshi [43] (SVM)	Anterior cruciate ligament tear detection in MRI images using multi-neighbor local binary pattern	India	MNLBP	Diagnosis	MRNet Dataset	339	No	89.41	88.00	92.00	-
Joshi [43] (KNN)	Anterior cruciate ligament tear detection in MRI images using multi-neighbor local binary pattern	India	MNLBP	Diagnosis	MRNet Dataset	339	No	88.92	87.00	92.00	-
Gupta [56]	Intelligent detection of knee injury in MRI exam	UAE	CNN	Diagnosis	MRNet Dataset	120	No	83.33	-	-	F1: 80.39
Jeon [57](China)	Interpretable and lightweight 3-D deep learning model for automated ACL diagnosis.	Singapore	CNN	Diagnosis	Radiologist	1177	No	-	95.00	94.50	AUC: 0.977
Jeon [57](MRNet)	Interpretable and lightweight 3-D Deep Learning Model For Automated ACL diagnosis.	Singapore	CNN	Diagnosis	Radiologist	1370	No	-	94.40	90.10	AUC: 0.963
Richardson [58] (NFS)	MR protocol optimization with deep learning: a proof of concept.	US	CNN	Diagnosis	Radiologist	200	No	-	93.00	99.30	AUC: 0.998
Germann [59]	Deep convolutional neural network-based diagnosis of anterior cruciate ligament tears: performance comparison of homogenous versus heterogeneous knee MRI cohorts with different pulse sequence protocols and 1.5-T and 3-T magnetic field strengths.	Switzerland	DCNN	Diagnosis	Arthroscopy	500	No	-	96.10	93.10	AUC: 0.961
Liu [60]	Fully automated diagnosis of anterior cruciate ligament tears on knee MR images by using deep learning.	US	CNN	Diagnosis	Arthroscopy	100	No	-	96.00	96.00	AUC: 0.98
Astuto [66]	Automatic deep learning-assisted detection and grading of abnormalities in knee MRI studies.	US	3DCNN	Classification	Radiologists	44	No	-	-	-	AUC: 0.9
Li [61]	Automated diagnosis of anterior cruciate ligament via a weighted multi-view network.	China	3DCNN	Diagnosis	Radiologist	208	No	-	-	-	AUC: 0.928

This table presents a summary of studies evaluating ACL diagnostic performance by clinicians and AI, with metrics to assess model effectiveness. Each entry lists the study author, dataset details, geographic origin, and backbone AI type (e.g., CNNs, deep learning). The study focus, either classification (grading cases, or categorising into partial, full or no tear) or diagnosis (detecting ACL injury), is noted under domain, while ground truth provides the validation standard, such as MRI. Sample size (*N*) and the application of radiomics features are indicated to contextualise the scale and approach. Key metrics include accuracy (overall correctness), sensitivity (true positive rate), and specificity (true negative rate), with other metrics offering additional insights (e.g., AUC, F1 Score, precision) for comprehensive performance comparison across studies

*DL* deep learning, *CNN* convolutional neural network, *DCNN* deep convolutional neural network, *TL* transfer learning, *FO-LOA* fractional-order snow leopard optimisation algorithm, *SVM* support vector machine, *MNLBP* multi-directional multi-neighbour local binary pattern, *IHBA* improved honey badger algorithm

### Risk of bias (RoB)

RoB and applicability assessments found most studies to be low risk: participants (91.7%), predictors (88.9%), outcomes (94.4%), and analysis (75.0%), with 75.0% overall low risk. High risk occurred in 8.3% of studies, all in the analysis domain. Unclear risk was seen in 16.7% overall, affecting participants (8.3%), predictors (11.1%), outcomes (5.6%), and analysis (16.7%). Applicability showed no high-risk ratings; unclear risk occurred in one study (2.8%) for predictors and outcomes. Most studies were low risk for applicability across participants (97.2%), predictors (94.4%), outcomes (94.4%), and overall (94.4%).

### Claim AI

Overall adherence to CLAIM-AI across the 36 studies was 70.1%. Compliance was highest for Criteria 2 (“Summary of study design, methods, results, and conclusions”) and Criteria 22 (“Detailed description of model”) at 100%, indicating strong reporting of methods and model details. Lowest adherence was for Criteria 12 (“How missing data were handled”) at 10.8%, Criteria 11 (“De-identification methods”) at 18.9%, and Criteria 18 (“Measures of inter- and intra-rater variability”) at 27%, highlighting gaps in reporting data handling, anonymisation, and annotation consistency (Fig. s3).

### Publication bias

For accuracy, there was no significant evidence of asymmetry (z = −0.48, *p* = 0.632). Similarly, for sensitivity, the test approached significance but did not reach conventional levels (z = −1.88, *p* = 0.060), though a warning regarding variance stability was noted. In contrast, for specificity, Egger’s test indicated strong evidence of funnel plot asymmetry (z = −5.59, *p* < 0.0001), suggesting possible publication bias in specificity estimates (Fig. s4).

### Systematic review

Among the reported models, 80.8% reported accuracy, 48.1% included sensitivity, and 73.1% included specificity as performance metrics. Chen et al achieved an accuracy of 99.47% [41], while Wang et al reported a diagnostic sensitivity and specificity of 98.0% [40], and Zhang et al demonstrated a specificity of 99.2% [52]. Five studies utilised radiomic models, employing various radiomic feature extraction methods [24–28] (Table s4). GT sources varied, with 19/52 (36.5%) derived from clinicians, 10/52 (19.2%) from arthroscopy, and 22/52 (42.3%) from prelabelled datasets, primarily KneeMRI (15/52, 28.8%) and MRNet (7/52, 13.5%), often without clearly specified ground truths.

The 12 studies (13 models) comparing AI to clinicians consistently demonstrated strong performance across accuracy, sensitivity, and specificity metrics, often surpassing clinician performance [27, 28, 37, 40, 41, 46, 47, 49, 59, 60, 62, 66]. Two studies compared AI to AI-assisted clinicians [37, 66], AI outperformed clinicians in 10 of the 12 studies in at least one metric, with Liu et al achieving equivalence with clinicians in sensitivity, specificity, and AUC at 96%, 96%, and 98%, respectively [60], while Xue et al provided precision data beyond the scope of this analysis [28]. Wang et al reported an AI accuracy of 91.84%, surpassing clinicians’ 80.5%, along with higher specificity (98.57% vs. 95.89%) [37]. Similarly, Chen et al reported AI achieving 97.5% accuracy, outperforming junior and mid-level trainees [41]. Of these studies, 7 (62.5%) compared AI with radiologists of varying levels [27, 47, 49, 59, 60, 62, 66], 2 (50%) with orthopaedic surgeons [37, 41], 2 (25%) comparing AI with groups of radiologists and orthopaedic surgeons [28, 46] and the remaining study (12.5%) comparing with radiologists and sports medics [40] (Table s4).

Eight studies (10 models) classified ACL tears as part of their diagnosis [26–28, 62–66]. Radiomic models were employed in three classification studies [26–28]. Seven classified injuries into full, partial, and no tear categories [26, 28, 62–66], while Chen et al graded tears from 0-IV, further classifying partial tears into stable and unstable as per the American Academy of Orthopaedic Surgeons [27, 67]. Classification models consistently reported key metrics, with 80.8%, 68%, and 65% reporting accuracy, sensitivity, and specificity, respectively. Notably, Xue et al [28] achieved a classification accuracy of 94.0% with an area under the receiver operating characteristic curve of 0.99, while Wang et al reported 96.8% accuracy and 100% sensitivity in differentiating ACL tear types [62].

The results of four classification-focused studies (five models) suggest an advantage of AI over clinicians [27, 28, 62, 66]. One study compared AI-assisted clinicians to standalone AI [66], while the remaining studies directly compared clinician and AI performance [27, 28, 62]. AI performance surpassed that of clinicians in at least one metric in all studies.

Five studies applied radiomics and machine learning to MRI for ACL tear diagnosis, using varied imaging sequences including T1WI, PDWI, PDW-SPAIR, and advanced fat-suppressed or thin-slice protocols [24–28]. All studies performed intensity normalisation, and four included data augmentation techniques to improve model generalisability [24, 26–28]. Feature extraction ranged from traditional first-order, shape, and GLCM-based metrics to deep learning-derived features, with feature selection methods such as LASSO, t-tests, and SelectKBest commonly applied. Segmentation approaches differed: three studies used manual annotation, one applied semi-automatic segmentation [25, 26, 28], and one employed fully automatic CNN-based segmentation [24].

### Meta-analysis

#### Overall diagnosis of ACL injuries

Figure 3 highlights a substantial range in diagnostic accuracy for ACL injuries, with values ranging from 55.00% [55] to 99.68% [64], with an overall pooled accuracy of 87.37% (84.31%, 90.43%). Sensitivity ranged from 65.09% [24] to a perfect 100% [39], resulting in a pooled sensitivity estimate of 90.73% (87.92%, 93.54%). Specificity spanned from 78.90% [55] to 99.61% [64], with a pooled estimate of 91.92% (90.07%, 93.77%). All findings had high heterogeneity (*p* < 0.01) (Fig. 2).

The ten classification models represented an overall pooled accuracy of 90.46% (85.50%, 95.41%), ranging from 86.33% to 99.68% [62, 64]. Sensitivity values varied from 80.53% to 97% [28, 62], yielding a pooled sensitivity estimate of 88.68% (83.78%, 93.58%). Specificity values range from 86.87% [26] to 99.62% [64], with a pooled specificity of 94.08% (91.04%, 97.12%) (*p* < 0.01) (Fig. 3).

#### Radiomic vs. non-radiomic models

Radiomic models demonstrated comparable or better performance in accuracy and specificity relative to non-radiomic models. Non-radiomic models achieved 86.95% (83.38%, 90.51%) accuracy, 91.22% (88.89%, 93.54%) specificity, and 92.23% (89.67%, 94.80%) sensitivity. Radiomic models showed slightly higher accuracy (87.26% (80.07%, 94.45%)) and specificity (93.03% (89.78%, 96.28%)), though sensitivity was lower (84.69% (70.77%, 98.62%)). However, none of these differences reached statistical significance, with overlapping CIs.

In classifying ACL tears, non-radiomic models showed strong diagnostic performance in ACL tear classification, with pooled accuracy (91.11% (84.94%, 97.28%)), sensitivity (89.51% (84.04%, 94.98%)), and specificity (94.45% (90.90%, 98.01%)). Among the three radiomic-based models analysed [26–28], two provided comparative data [26, 28]. Xue et al’s model reported 97% sensitivity and specificity [28], outperforming the non-radiomic models’ benchmarks, while Dung et al’s model reported lower values (87.87% accuracy, 83.68% sensitivity, 88.92% specificity) than non-radiomic benchmarks [26].

#### Diagnosis of ACL injuries by clinician vs. AI

AI models showed comparable performance to clinicians for ACL tear detection, although individual study outcomes varied. Pooled SMDs indicate a slight AI advantage in specificity (0.52 (−0.31, 1.36)) and sensitivity (0.19 (−0.47, 0.84)), with sensitivity values ranging from −2.15 to 2.59 [47, 49], but these differences were not statistically significant, as the 95% confidence intervals included zero. The pooled SMD for accuracy is 1.51 (0.61, 2.40), ranging from −1.23 to 3.70. When compared to radiologists, AI showed equivalent accuracy (1.41 (−1.23, 4.06)), sensitivity (0.31 (−0.54, 1.16)), and specificity (0.56 (−0.79, 1.91)), though none of these differences were statistically significant, as all confidence intervals crossed zero. Considerable heterogeneity was noted across these comparisons (I² = 98.96%).

In classification accuracy, SMD values range from 0.11 [28] to 3.12, with the pooled accuracy being 1.53 (0.47, 2.58) [62]. Sensitivity SMD values ranged from −0.76 to 2.03, with an overall pooled SMD of 0.67 (−1.26, 2.60) [62], reflecting non-significant findings due to wide CIs. Specificity SMD values ranged from 0.77 to 1.42, with a pooled SMD of 1.34 (1.08, 1.60) [27, 62]. This suggests a consistent advantage of AI models over clinicians in specificity, with low heterogeneity indicating relatively stable specificity results compared to other metrics (*p* = 0.36) (Fig. s10).

#### Diagnosis of ACL injuries by MRI slice thickness and sequence

PDFS sequences outperformed Proton Density (PD) sequences, achieving numerically higher accuracy and specificity (Figs. s11, s12). PDFS models reported 90.50% (87.87%, 93.13%) specificity, peaking at 99.2% with sagittal, coronal, and axial images using 1.5-T and 3-T scanners [52]. Sensitivity averaged 90.43% (87.04%, 93.82%), with a maximum of 98% using sagittal and coronal images [40]. Accuracy for this group was 86.16% (82.14%, 90.09%), peaking at 99.6% [40]. PD models (without FS) had slightly lower accuracy (85.11% (81.24%, 88.98%), max 99.47%) and specificity (89.59% (86.80%, 94.73%), max 98.84%), despite overlapping Cis [41]. Sensitivity averaged 90.77% (85.49%, 96.84%), with 100% sensitivity using coronal images on 1.5-T and 3-T scanners [48].

2D sequences achieved a pooled 90.77% (86.80%, 94.73%) sensitivity, and 91.69% (89.77%, 93.61%) specificity, but were outperformed by Namiri et al, who used a proton density-weighted 3D fast spin-echo (CUBE) sequence [65]. Due to an insufficient sample size using solely 3D sequences, no meta-analysis was possible.

Slice thickness influenced diagnostic performance. Slices of < 2 mm achieved the highest sensitivity (94.47% (91.98%, 96.95%)) and specificity (94.27% (91.45%, 97.09%)), whereas ≥ 3 mm slices had slightly lower sensitivity (92.53% (87.96%, 97.10%)) but the highest accuracy (88.34% (84.71%, 91.98%)). However, overlapping CIs across slice thickness subgroups suggest that these differences may not be statistically significant and should be interpreted cautiously.

Of the classification models, due to limited sample sizes, a meta-analysis was performed exclusively for models using PDW-based sequences. 1.5-T FS-PDWI achieved the highest accuracy (99.68%) and specificity, surpassing the pooled accuracy (90.07% (83.75%, 96.39%)) and specificity (93.99% (90.40%, 97.50%)) [64]. Sensitivity peaked at 97% using 3-T PDW-SPAIR, exceeding the pooled sensitivity of 88.18% (82.49%, 93.86%) [28]. However, overlapping CIs suggest these comparisons should be viewed as descriptive rather than conclusive.

#### Diagnosis of ACL injuries by ground truth

AI models using Arthroscopy as the GT had the highest accuracy (93.09% (89.82%, 96.36%)) sensitivity (94.17% (91.21%, 97.13%)), and specificity (93.57% (91.06%, 96.08%)), followed by clinicans as the GT. While these values suggest a performance advantage when clinical or surgical confirmation is used, the differences between subgroups are modest and confidence intervals overlap, limiting definitive conclusions.

Most studies used radiologists as the GT, with the best-performing models using radiologists [65], orthopaedic surgeons [37], or a combination of both as the GT [41], with the best performance reported by Chen et al, who used a combination of senior, mid-level, and junior trainees, each group included orthopaedic surgeons and radiologists.

## Discussion

This meta-analysis, synthesising data from 36 studies, provides a comprehensive evaluation of AI in ACL tear diagnosis and classification. Overall, AI achieved high accuracy (87.37% (84.31%, 90.43%)), sensitivity (90.73% (87.92%, 93.54%)), and specificity (91.34% (89.17%, 93.52%)), supporting its clinical utility. For ACL tear grading, the ten classification models demonstrated pooled accuracy of 90.46% (85.50%, 95.41%), sensitivity of 88.68% (83.78%, 93.58%), and specificity of 94.08% (91.04%, 97.12%).

There is a notable lack of high-level evidence, such as meta-analyses, on the use of radiomics for diagnosing ACL tears. In other orthopaedic knee contexts, radiomics has demonstrated strong diagnostic performance, underscoring its potential to outperform traditional diagnostic methods [68–70]. Although these trends are not specific to the ACL, they underscore the broader potential of radiomics to outperform traditional diagnostic methods. This generalisable strength suggests that, when applied to ACL imaging, radiomics, especially when integrated with DL, could yield more robust and clinically useful models for tear assessment. This could be achieved by radiomic-based analysis identifying subtle variations in ligament texture, shape, and surrounding tissue through handcrafted features [21, 71]. While DL models require large datasets and may miss clinically relevant nuances, radiomics can detect early or partial tears by quantifying fine image patterns such as intensity or heterogeneity. This is especially important in ACL imaging, where abnormalities may be subtle. Radiomic pipelines also allow for targeted feature selection and greater interpretability, making them well-suited for smaller datasets and clinical settings where transparency is essential. As a result, radiomic models may offer more accurate and clinically meaningful assessments of ACL tears than end-to-end DL systems and integrated into DL systems. Future research is needed to identify optimal radiomic features and validate their performance across platforms, as reliability and prognostic value are highly dependent on the extraction method and feature set [72].

The superior diagnostic performance observed in models trained using specific imaging sequences, such as the notably high accuracy (99.68%) and specificity achieved using 1.5-T FS-PDWI, and the peak sensitivity of 97% with 3-T PDW-SPAIR, may be partially explained by differences in magnetic field strength. Higher field strengths, such as 3 T, are associated with longer T1 relaxation times, which enhance soft tissue contrast and lesion conspicuity, potentially improving tear detection [73–76]. Additionally, these biophysical factors, including magnetic field strength, along with sequence optimisation and slice thickness, may have contributed to the observed performance differences. Thicker slices improve signal-to-noise ratio by increasing voxel volume and capturing more signal, which can enhance overall image quality. However, this comes at the cost of reduced spatial resolution and greater partial volume effects, which may obscure fine anatomical details that are critical for detecting subtle or partial ACL tears [77, 78]. These MRI trade-offs may help explain the variability in performance by sequences and slice depth, although such interpretations remain speculative given the limited size and heterogeneity of the available subgroup data. From a practical standpoint, these findings underscore the importance of standardised imaging protocols when training and deploying AI models for ACL assessment. Minor variations in slice thickness or field strength could significantly influence performance, especially when detecting partial or subtle tears. As AI tools move closer to clinical use, understanding these technical dependencies becomes critical for implementation and quality assurance.

AI, as a supportive tool, can reduce false negatives and provide valuable adjudication, particularly benefiting less experienced clinicians who can leverage AI as an adjudicator to refine their skills and reduce diagnostic errors, aligning with our findings [79, 80]. This adjudication layer minimises unnecessary surgeries and prevents delays in necessary procedures. Integrating AI into radiology training enhances image reporting quality and efficiency, addressing the increasing demands of modern healthcare [81]. By enabling faster, more reliable analysis and flagging potential issues, AI improves diagnostic workflows. Even the same radiologist may demonstrate varying sensitivity and specificity depending on the prevalence of the findings being assessed; in such contexts, specificity becomes especially critical when identifying rare or subtle abnormalities. Here, AI can serve as a valuable support tool to enhance specificity and reduce missed uncommon findings in everyday clinical workflows. However, using AI as a standalone decision-maker without human oversight is premature, as it would require robust, fail-safe validation standards that currently do not exist, making full delegation of diagnostic responsibility to AI not presently feasible.

Duong et al propose that AI integration in training and clinical practice can standardise diagnostic quality, provide consistent, evidence-based feedback to junior clinicians, and reduce variability [82]. This approach not only aids training but also enhances patient outcomes by improving the accuracy and consistency of radiological reporting [83]. While triage systems using AI have shown success in acute scenarios such as intracranial haemorrhage detection in emergency settings, extrapolating this model to musculoskeletal cases like ACL injuries requires caution. Although earlier surgical intervention for ACL tears can improve outcomes, these injuries generally do not carry the same urgency or morbidity as acute neurological emergencies [84]. Moreover, the apparent simplicity of imaging findings does not always reflect the clinical complexity or functional impact of the injury. Given the growing interest in conservative management of certain ACL injuries, the clinical relevance of AI-based classification lies in its ability to inform prognosis and align with patient-specific treatment pathways rather than rigid morphological categories alone [67, 85]. There may also be ACL imaging features predictive of suitability for conservative treatment, potentially derived through radiomic feature extraction, that are not captured in current classification frameworks. Future work should explore these possibilities and integrate patient-specific variables and outcomes to supervise training, ensuring that AI tools support nuanced clinical decision-making. Successful adoption will depend not only on diagnostic performance but also on adaptability to clinical workflows. Embedding AI within radiology infrastructure such as PACS and reporting systems, with human oversight, will be essential for safety, trust, and usability. Implementation should prioritise workflow compatibility, clinician engagement, and real-world validation to translate algorithmic advances into sustained clinical benefit, with the same principles guiding industry-led radiology interface development from the outset to ensure practical integration, transparency, and clinical relevance.

The variability in sequences across studies raises concerns about model generalisability in clinical practice [48, 52, 65]. Even minor differences in scanner manufacturers, imaging protocols, and field strengths can affect model performance, potentially leading to diagnostic inconsistencies. While differentiating full-thickness from partial ACL tears demonstrates technical advancement, the clinical relevance of such distinctions remains complex. Subtotal tears, often labelled as partial, may still cause functional instability and ultimately require surgical intervention. With increasing interest in non-operative management, it is essential to evaluate whether AI-based classifications reflect real-world treatment decisions and correlate with clinical outcomes [86–88]. Most included models focused on anatomical grading using binary (“tear” vs. “no tear”) or categorical (“partial”, “complete”, or “intact”) classifications, yet few assessed prognostic validity or clinical utility. Notably, of the models classifying ACL tears, only Chen et al used a validated scale, the American Academy of Orthopaedic Surgeons grading system [27, 67]. While newer tools like the ACL Injury Severity Scale (ACLISS) assess post-reconstruction tissue damage, no widely adopted classification system exists for ACL tears [89].

In practical terms, AI can streamline workflows by pre-analysing images and providing preliminary diagnoses for clinicians to verify, reducing case time and allowing clinicians to focus on complex decision-making [90, 91]. This collaborative model, where AI supports rather than replaces clinical judgement, enhances reporting efficiency and improves volume and quality while emphasising ethical and transparent AI use [92]. However, the impact of AI inference bias, the human tendency to agree with AI-generated conclusions, on clinical reporting remains underexplored, requiring further research on its impact on clinical decision-making [93]. A related concern is that over-reliance on AI may shift radiology training away from active image interpretation toward passive verification of AI outputs. This risks a gradual erosion of core interpretive skills, with radiologists potentially becoming reviewers of AI-generated reports rather than independent diagnostic experts. Therefore, AI should not be viewed as a replacement for rigorous clinical training or serve as a crutch for inadequately trained radiologists, but rather function as a supportive tool that enhances the expertise of well-trained clinicians. Additionally, regulatory frameworks such as the EU AI Act aim to safeguard patient safety by distinguishing ethically approved model development from clinical deployment [94]. While some have expressed concern that such regulations may constrain adaptability, the Act does not prohibit model training or evolution [95, 96]. Continued model training and refinement can proceed within protected research environments, supporting innovation without compromising patient safety. Transparent validation, regulatory-aligned update pathways, and federated learning strategies should be prioritised to balance AI performance with clinical accountability.

Despite its potential, the limited interpretability of AI models remains a major obstacle to clinical integration and trust, as the “black box” nature of these systems obscures the reasoning behind their decisions [97]. Currently, few models provide insight into decision-making processes, highlighting the need for greater transparency and interpretability in future AI systems [98]. Additionally, significant heterogeneity across studies underscores the importance of standardised reporting practices, including consistent interobserver agreement metrics, to enhance model quality [66]. Further validation using diverse scan types and alternative [84] GTs, including arthroscopic data and follow-up outcomes, would improve external validity. AI could also evolve to predict surgical outcomes using imaging biomarkers, facilitating patient stratification for optimal management.

Our systematic literature search enabled a robust pooled analysis, but several methodological limitations warrant caution. Many studies simplified ACL tear classification into broad categories such as intact, partial, or full tears, limiting diagnostic nuance, and for meta-analysis, these were treated as diagnostic categories with classification considered a subgroup. Inconsistent reporting of outcome measures led us to rely on accuracy, sensitivity, and specificity as primary metrics. Data-related issues, including class imbalance, small or poor-quality datasets, and missing confidence intervals estimated via binomial assumptions, may have introduced bias. GT definitions varied, with some studies using prelabelled datasets without clear clinician involvement, while arthroscopy-based GTs may capture more overt imaging findings, and surgeon–radiologist consensus may better reflect real-world interpretation, potentially introducing sampling bias. Detailed stratification by lesion type, such as isolated partial tears, was rare, and direct comparisons between AI and radiologists at this level were largely absent, preventing assessment of which lesions were most challenging for AI or how this compared to clinician performance. Prevalence-related variability also affects accuracy, which remains sensitive to underlying distributions despite stratified train-test splits, and the absence of full confusion matrices further limits evaluation of diagnostic metrics. Mixed 1.5-T and 3-T MRI data precluded field strength comparison, while limited subgroup sample sizes reduced statistical power and often produced wide confidence intervals, leading us to avoid formal statistical testing to prevent overinterpretation. Despite methodological safeguards, these limitations highlight the need for cautious interpretation and emphasise the importance of standardised reporting in future research.

Future research should prioritise the development of a standardised grading system to improve AI training and diagnostic consistency, with particular emphasis on classifying partial ACL tears into stable and unstable grades. These cases often present subtle imaging features and diagnostic ambiguity, making targeted efforts with well-balanced and representative datasets essential to enhance model performance and clinical decision-making. Incorporating widely used clinical outcome measures, such as the International Knee Documentation Committee (IKDC) satisfaction form, Lysholm score, and Tegner activity scale, along with indicators like failed trials of conservative treatment, could help bridge the gap between imaging-based classifications and real-world treatment decisions. In addition to technical factors, variations in patient demographics such as age, sex, and ethnicity complicate the generalisability of AI models, potentially introducing patient-level biases that limit broader applicability [99]. Observed variation in GTs outlines potential discrepancies in model performance across datasets like KneeMRI and MRNet [16, 49]. Moreover, variations in GTs, whether based on arthroscopic findings or clinician interpretations, pose challenges in achieving standardisation. Particular emphasis should be placed upon the validation of AI models across MRI systems and patient cohorts, prioritising arthroscopic records as the gold standard to mitigate biases from imaging equipment and demographics.

## Conclusion

AI models demonstrate diagnostic performance comparable to clinicians and may serve as a valuable adjunct in ACL tear detection, pending further validation in prospective clinical settings. Radiomics-based models and a novel standardised classification system are key to future development. Validation across diverse GTs and imaging protocols will ensure broader applicability, making AI a valuable tool for enhancing diagnostic accuracy and clinical workflows.

## Supplementary information

ELECTRONIC SUPPLEMENTARY MATERIAL

## Abbreviations

2D: Two-dimensional
3D: Three-dimensional
ACL: Anterior cruciate ligament
AI: Artificial intelligence
AUC: Area under the curve
CLAIM-AI: Checklist for artificial intelligence in medical imaging
CNN: Convolutional neural network
DL: Deep learning
F1: F1 score
FS: Fat suppressed/fat suppression
GT: Ground truth
IHBA: Improved honey badger algorithm
MRI: Magnetic resonance imaging
PD: Proton density
PDFS: Proton density fat suppression
PDW: Proton density-weighted
PRISMA: Preferred Reporting Items for Systematic Reviews and Meta-Analyses
PRISMA-P: Preferred Reporting Items for Systematic Reviews and Meta-Analyses Protocols
R: R programming language
RoB: Risk of bias
ROI: Region of interest
SMD: Standardised mean difference
SPAIR: Spectral attenuated inversion recovery
SVM: Support vector machine
T: Tesla
