# Supplementary material for: AI demonstrates comparable diagnostic performance to radiologists in MRI detection of anterior cruciate ligament tears: a systematic review and meta-analysis
Source: Eur Radiol. 2025 Sep 25;36(4):2500–17. doi: 10.1007/s00330-025-12020-2 (PMC13035667; doi:10.1007/s00330-025-12020-2)
Supplement: Supplementary file 1 — ELECTRONIC SUPPLEMENTARY MATERIAL [file 330_2025_12020_MOESM1_ESM.pdf]

AI Demonstrates Comparable Diagnostic Performance to Radiologists in MRI Detection of Anterior Cruciate Ligament Tears: A Systematic Review and Meta-Analysis

ELECTRONIC SUPPLEMENTARY MATERIAL

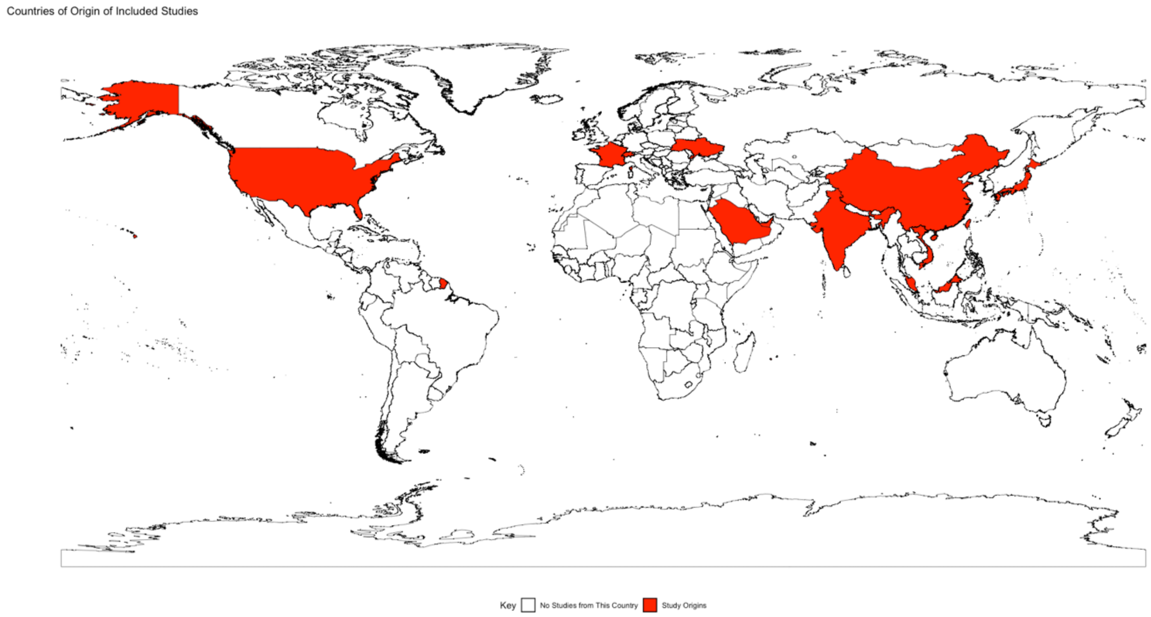

Figure s1 – World Map
This world map illustrates the countries of origin for studies included in the systematic review. Countries highlighted in red represent those from which relevant studies were sourced, while countries in white had no studies included in this analysis. All data visualization was generated in R using the ggplot2, rnaturalearth, rnaturalearthdata, and dplyr packages. The rnaturalearth, rnaturalearthdata and rnaturalearthhires
packages provided the geographic data, while dplyr was used for data manipulation, and ggplot2 was utilized to create the map visualization.

Eur Radiol (2025) Gill SS, Haq T, Zhao Y, Ristic M, Amiras D, Gupte CM.

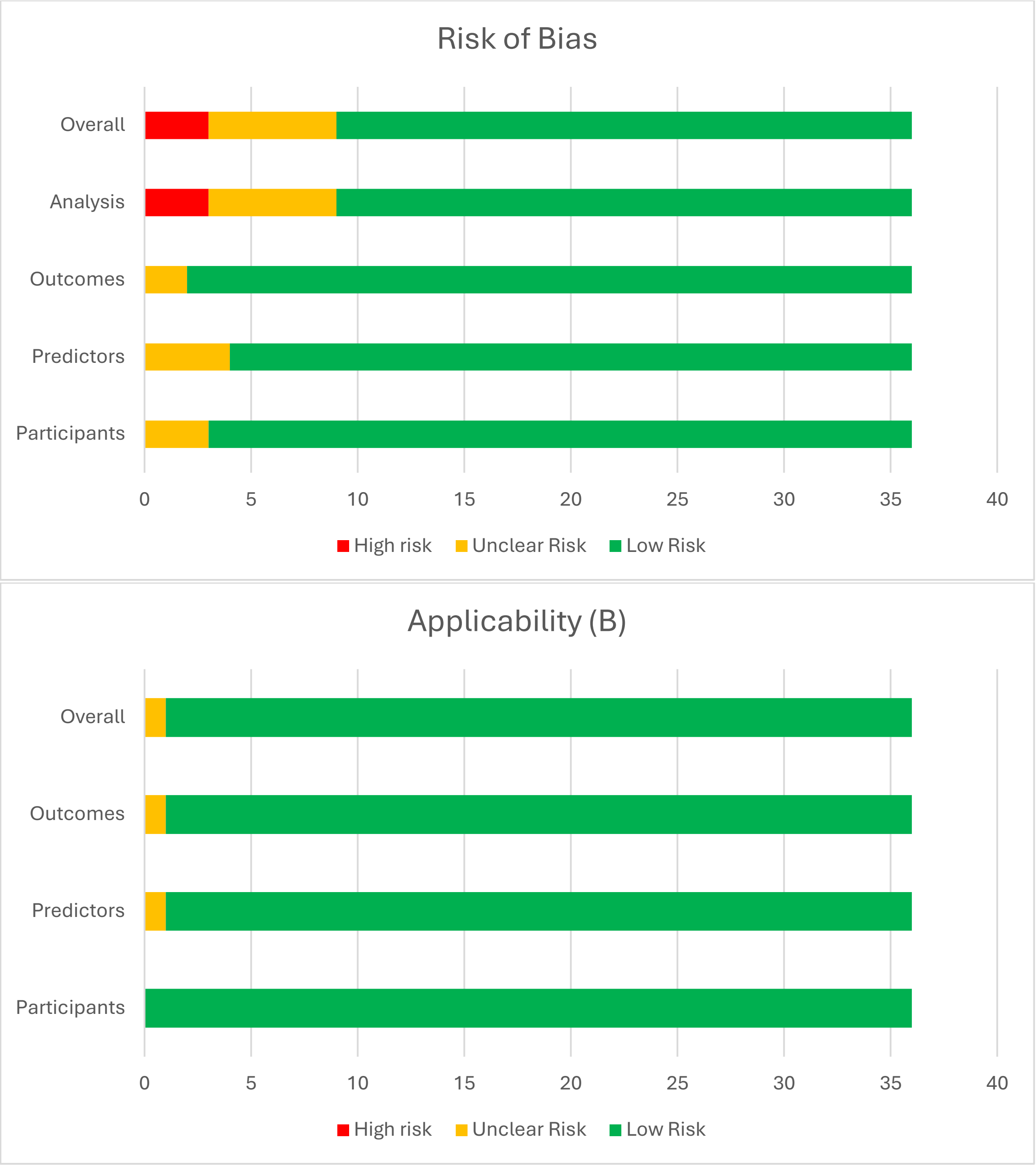

Figure s2: PROBAST Risk of Bias Analysis
(A)Analysis of Risk of Bias; (B)Analysis of the applicability of studies.

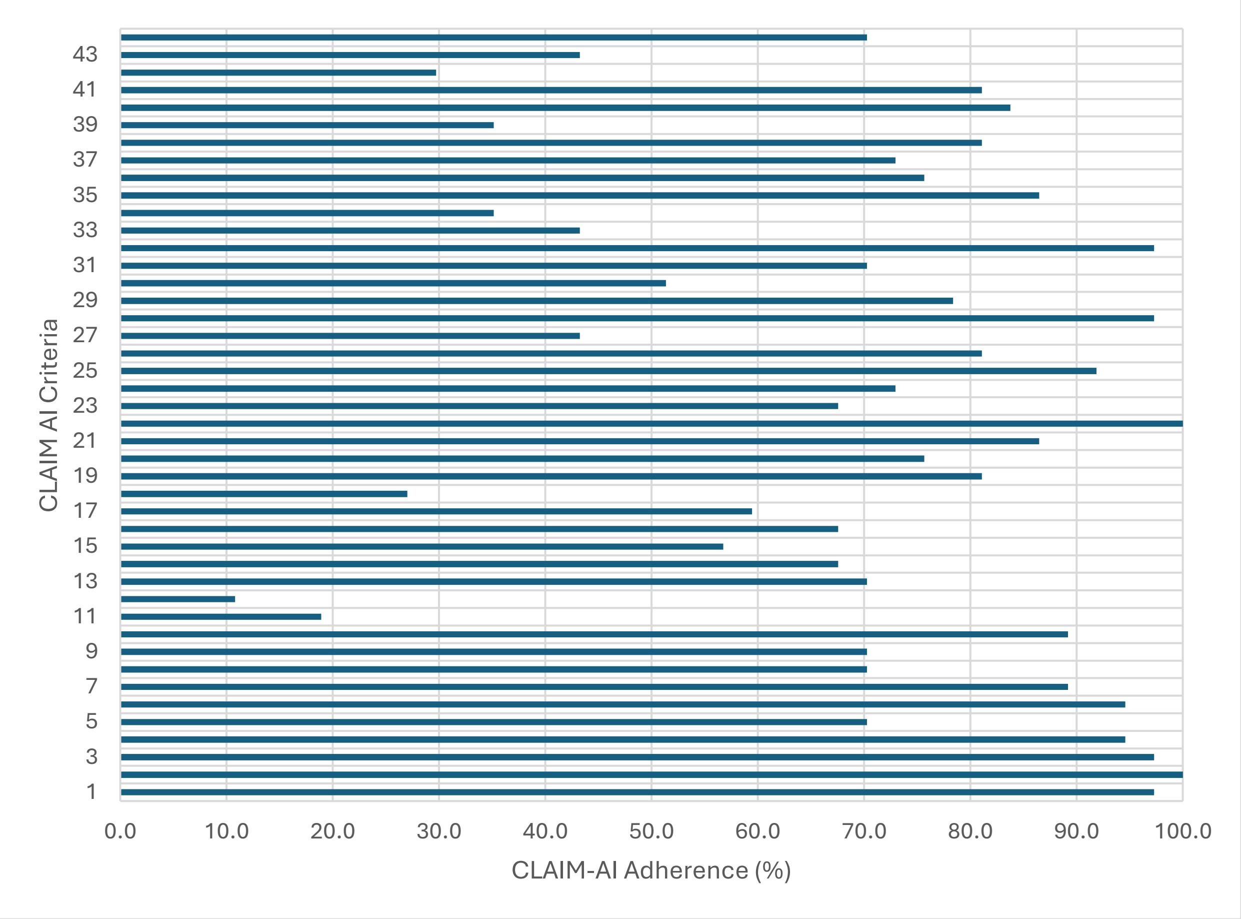

Figure s3: CLAIM-AI Summary

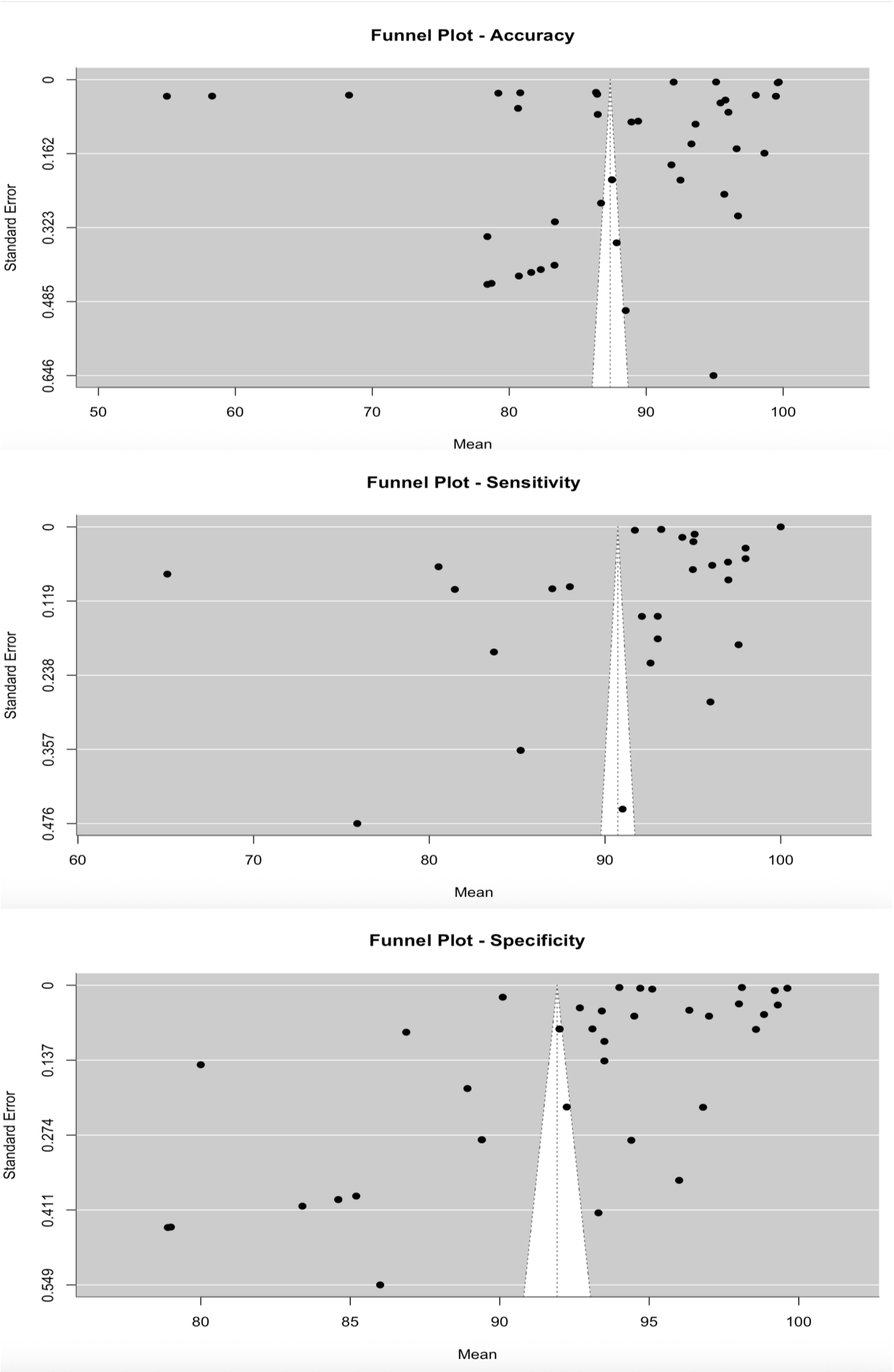

Figure s4: Funnel plots

Eur Radiol (2025) Gill SS, Haq T, Zhao Y, Ristic M, Amiras D, Gupte CM.

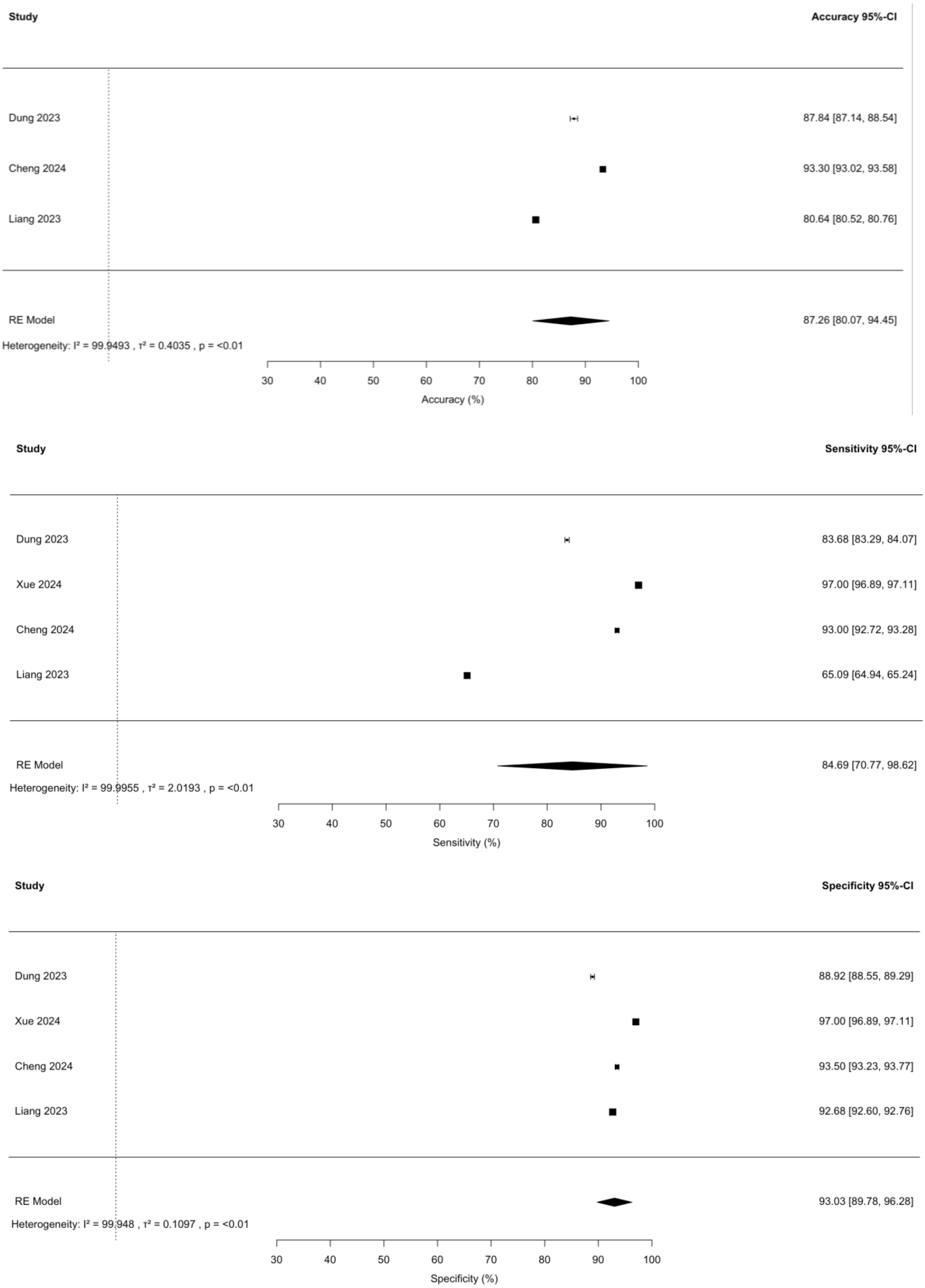

Figure s5: Forest Plot of Outcomes of AI's Performance in Diagnosing ACL Tears by Radiomic AI Models

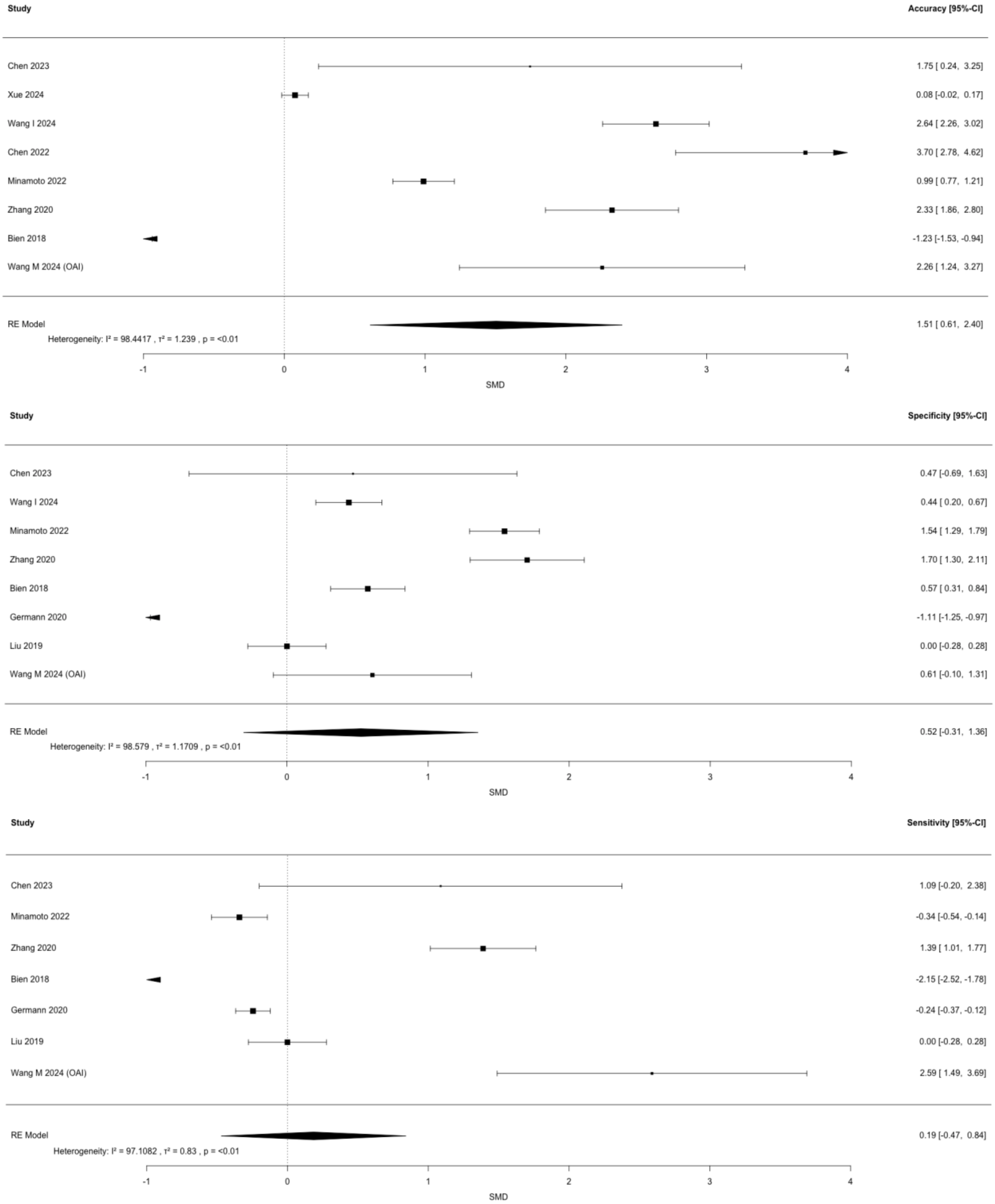

Figure s6*: Forest Plot of Outcomes Comparing AI and Clinicians in Diagnostic Models*

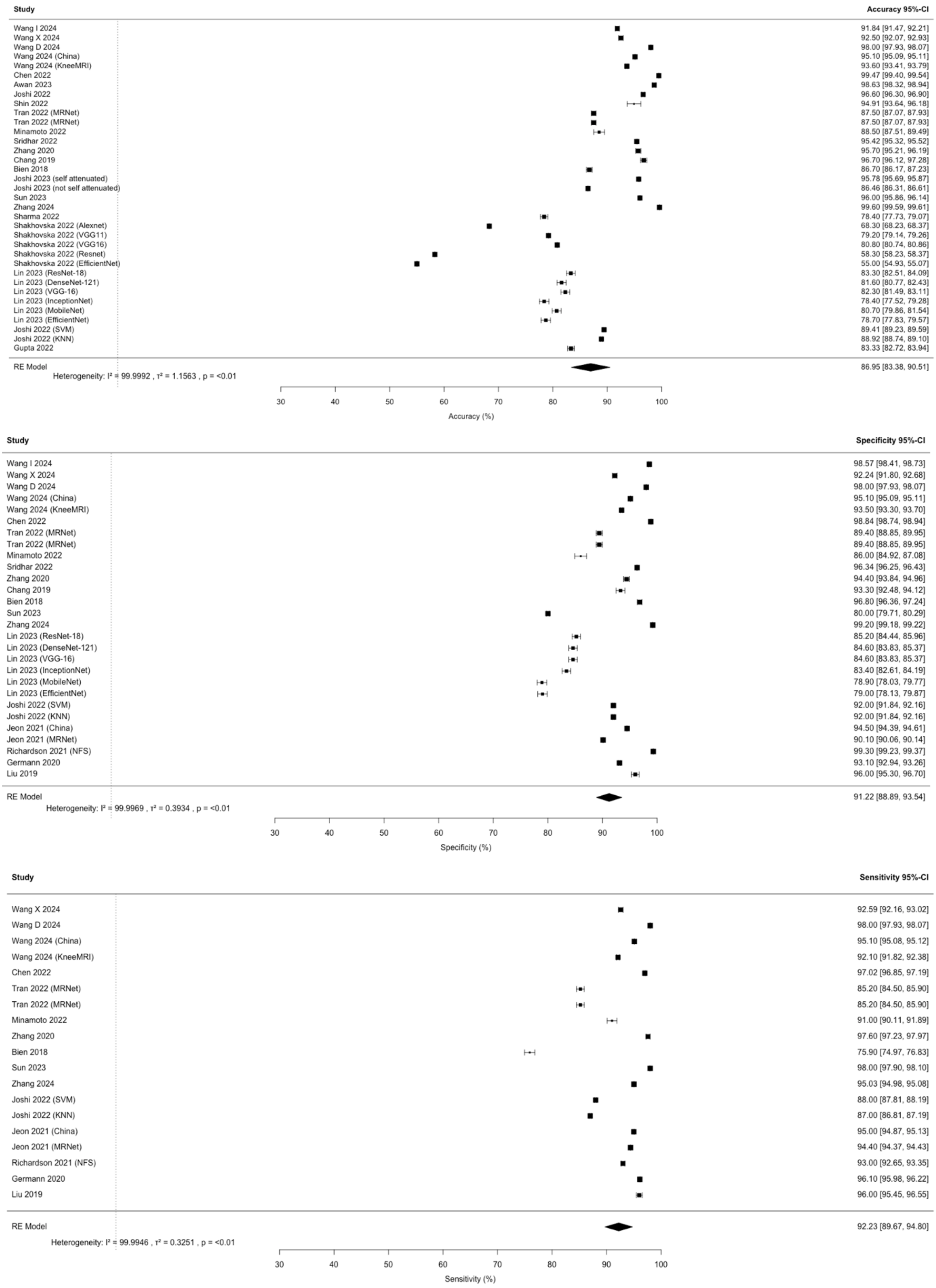

Figure s7: Forest Plot of AI's Performance in Diagnosing ACL Tears by Non-Radiomic AI Models

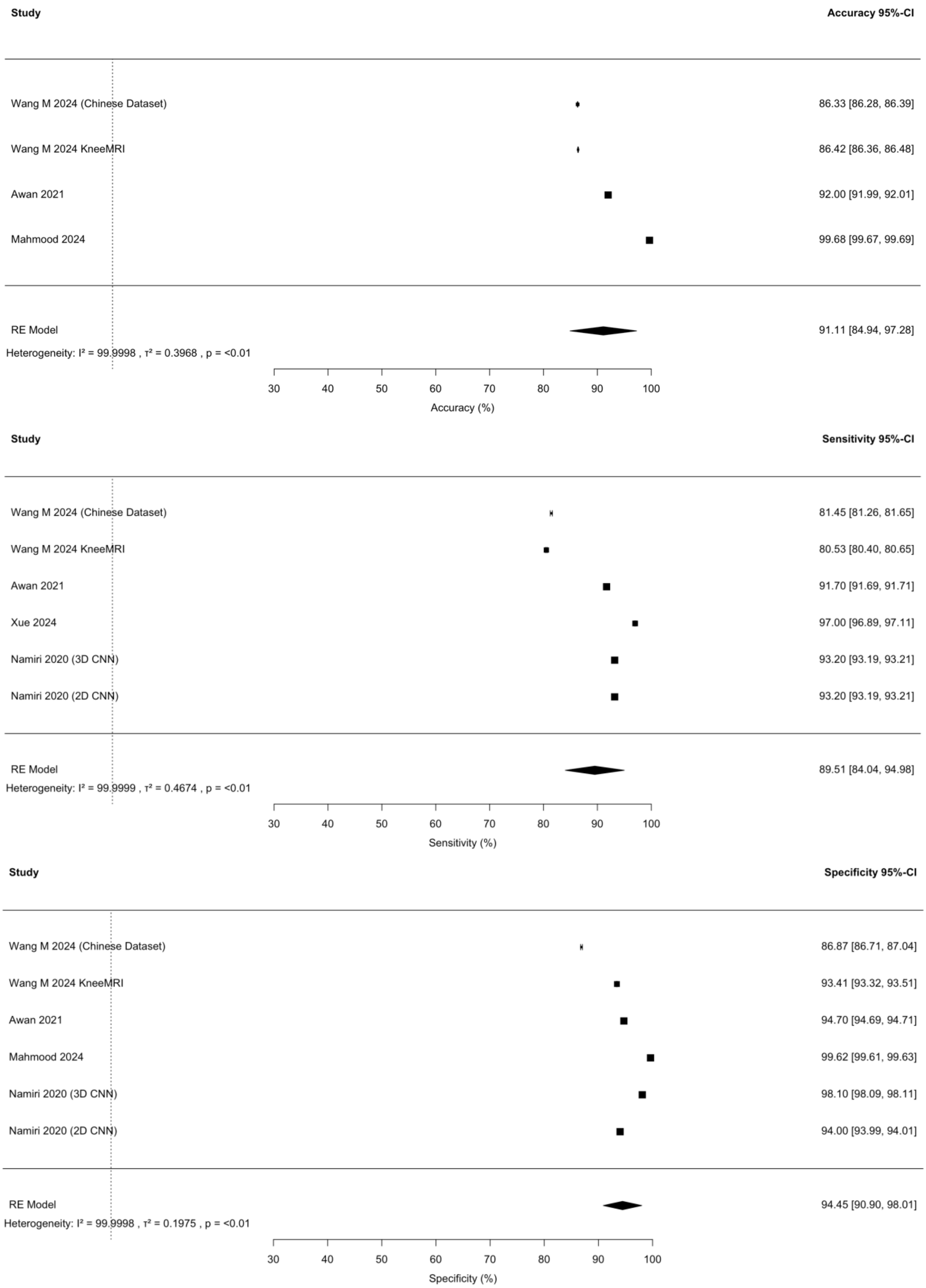

Figure s8: Forest Plot of of AI's Performance in Classifying ACL Tears by Non-Radiomic AI Models

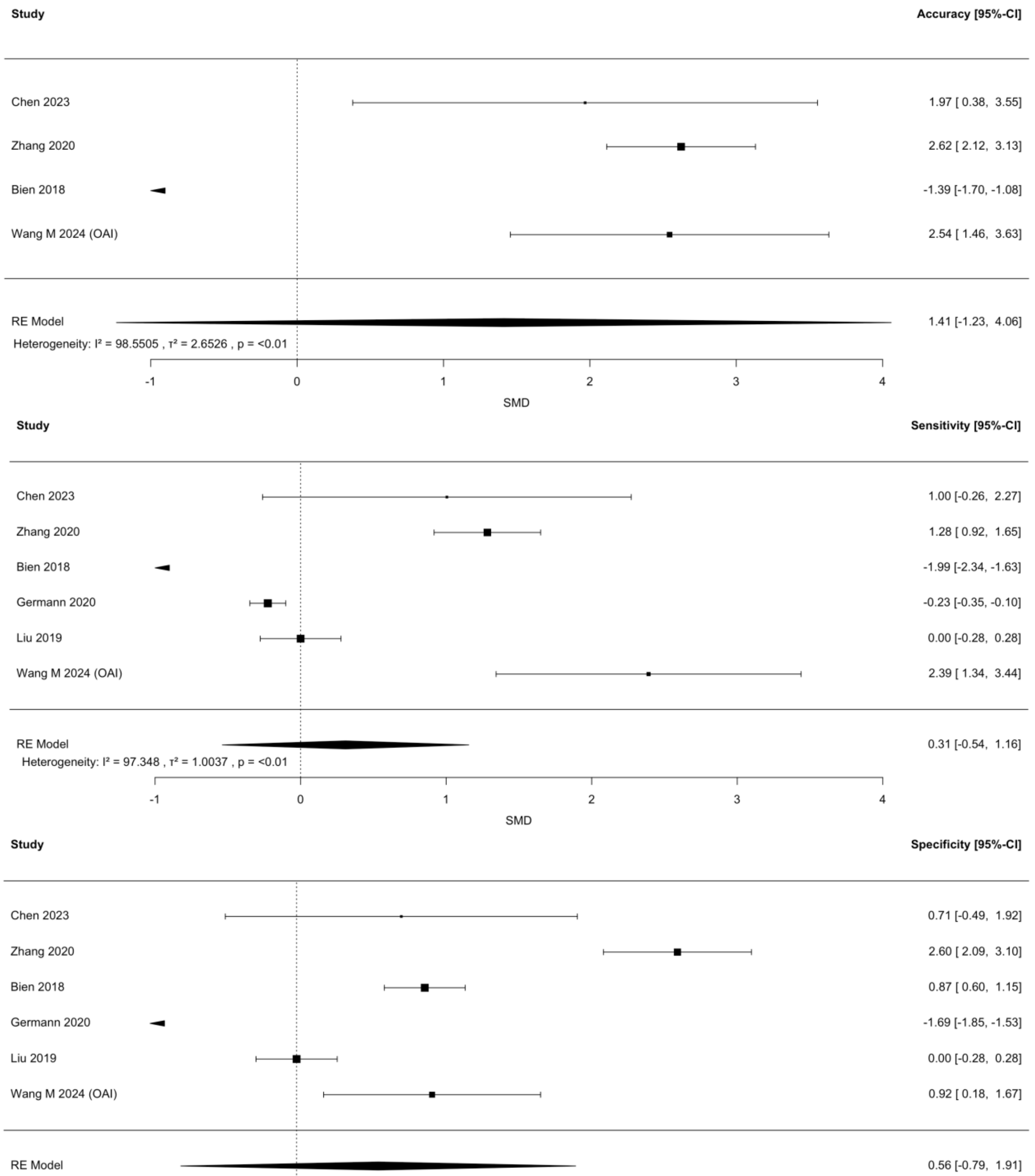

Figure s9: Forest Plot of Comparisons between AI and Radiologists in Diagnostic Model

Values greater than 0 favour AI over clinicians.

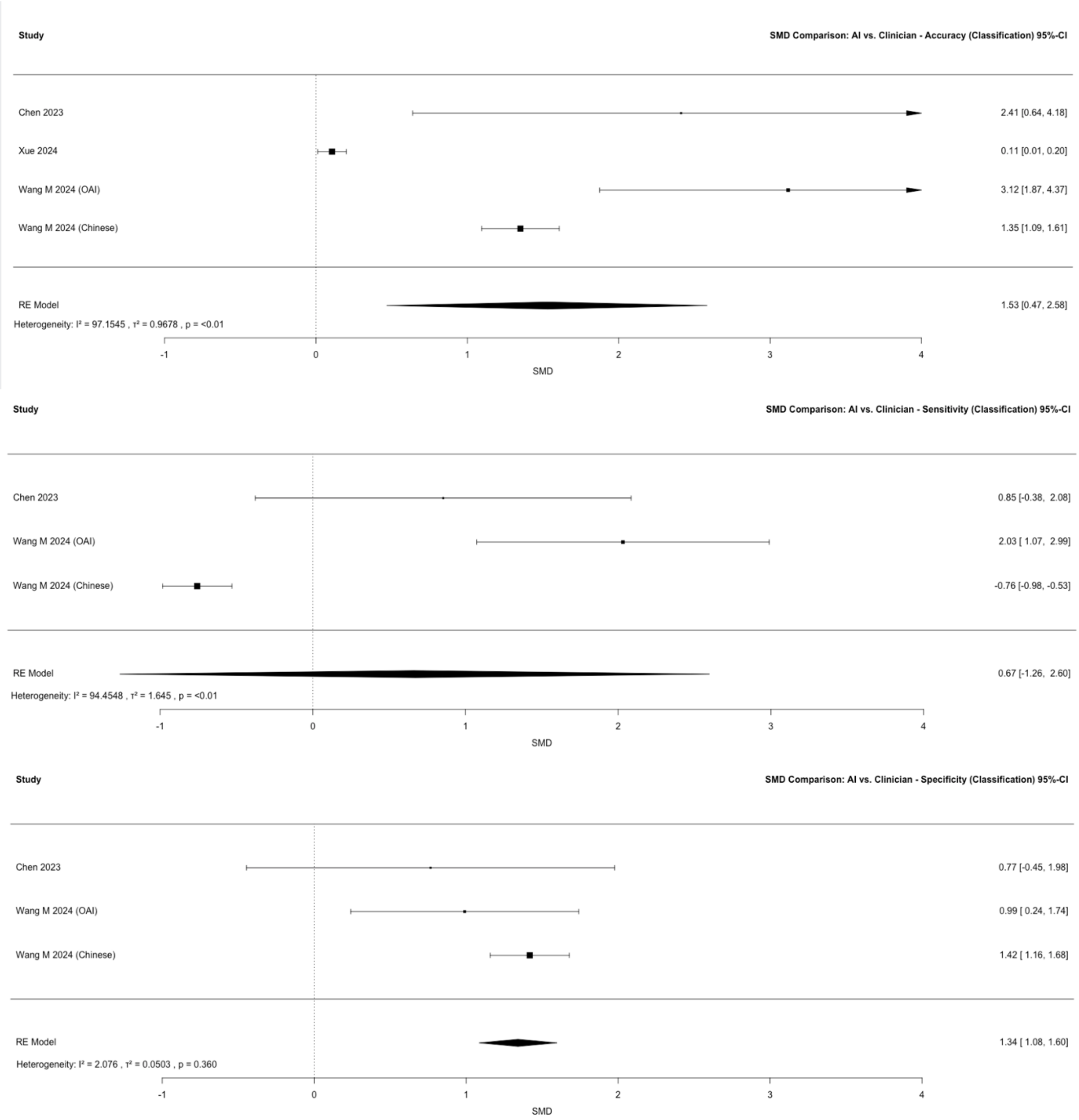

Figure s10: Forest Plot of Comparisons between AI and Clinicians in Classification Models

Values greater than 0 favour AI over clinicians.

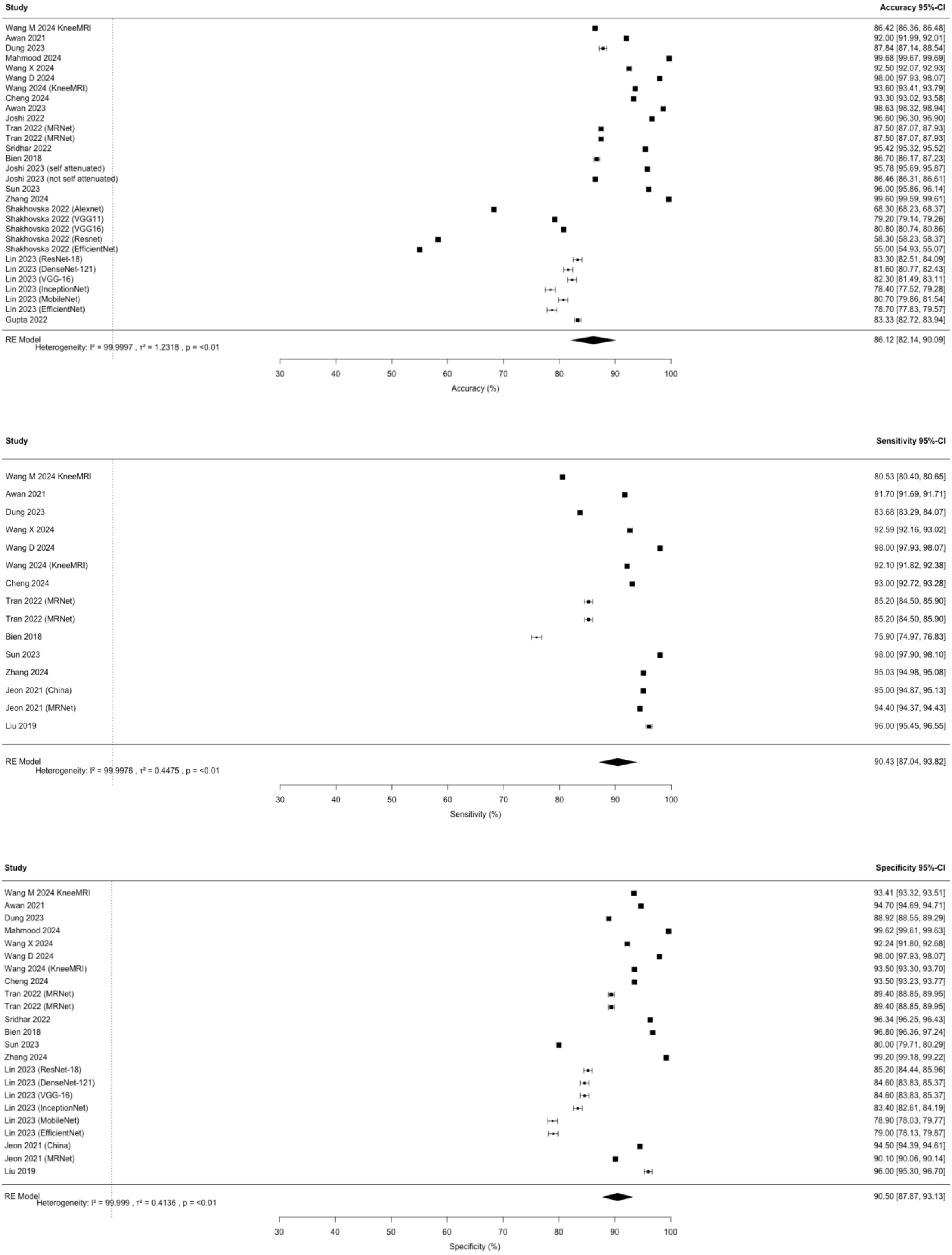

Figure s11: Forest Plot *of Outcomes of* AI using PDFS MRI Sequences

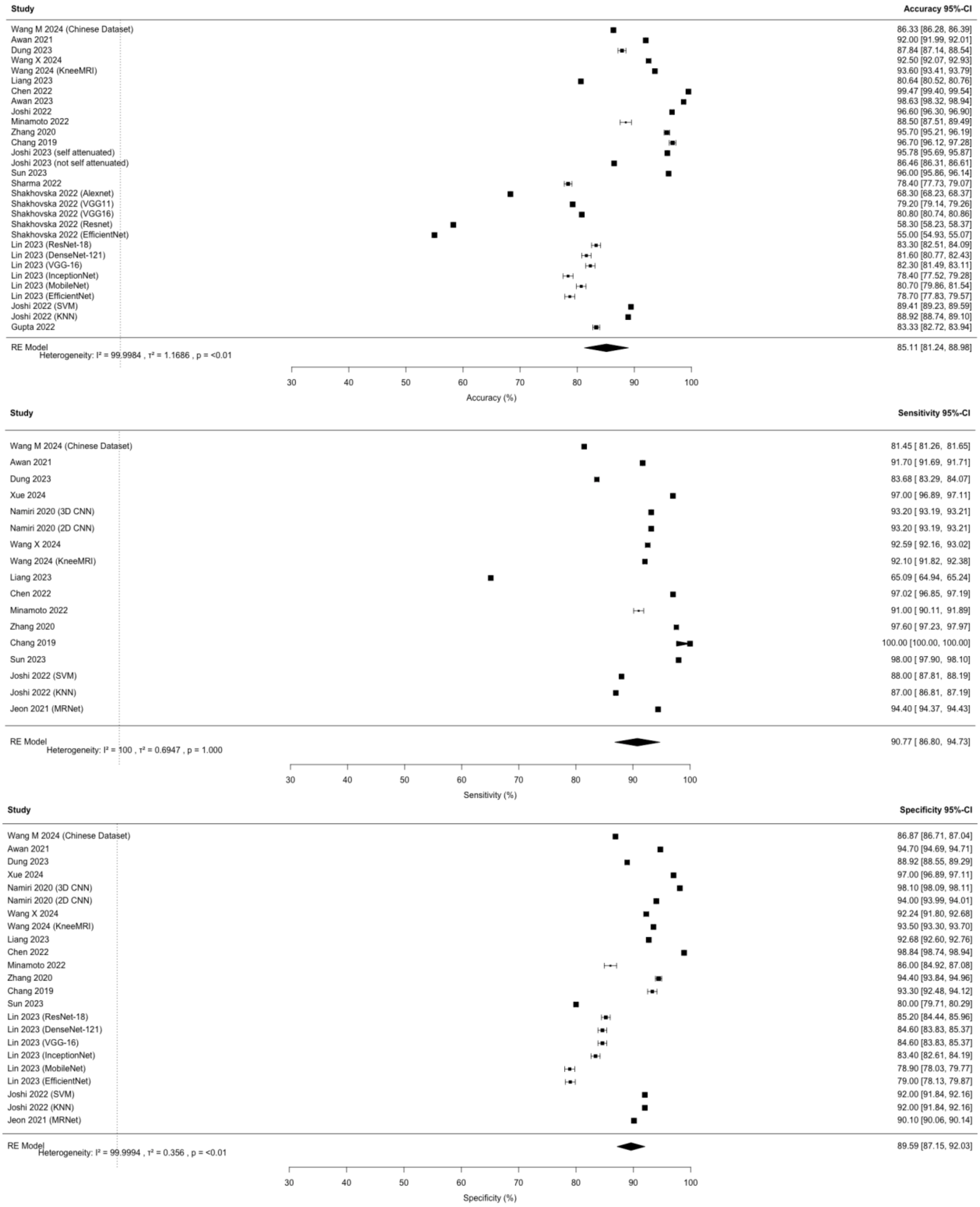

Figure s12: Forest Plot *of Outcomes of* AI using PD MRI Sequences

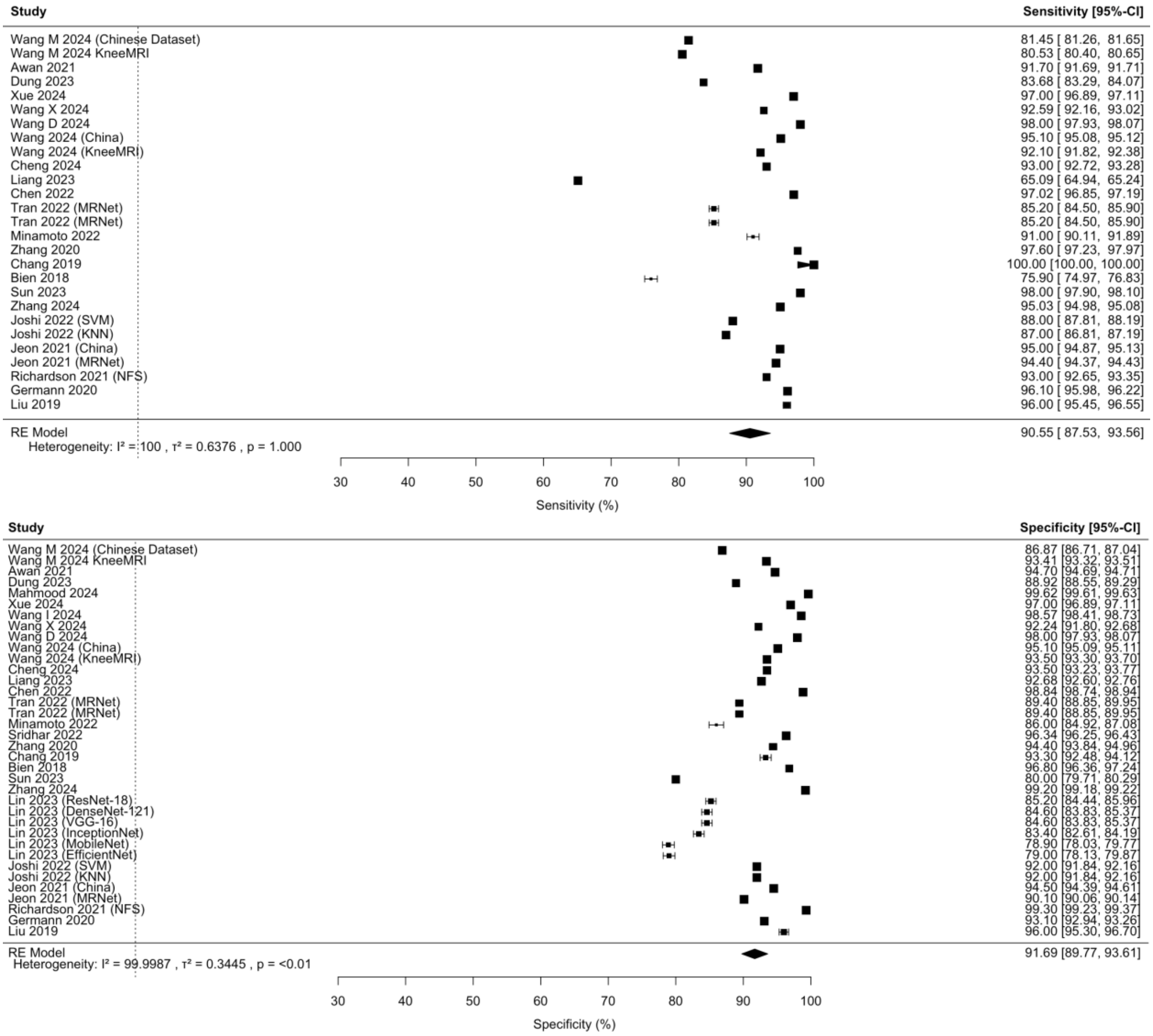

Figure s13: Forest Plot of Outcomes of Diagnosis of ACL tears Using 2D MRI Sequences

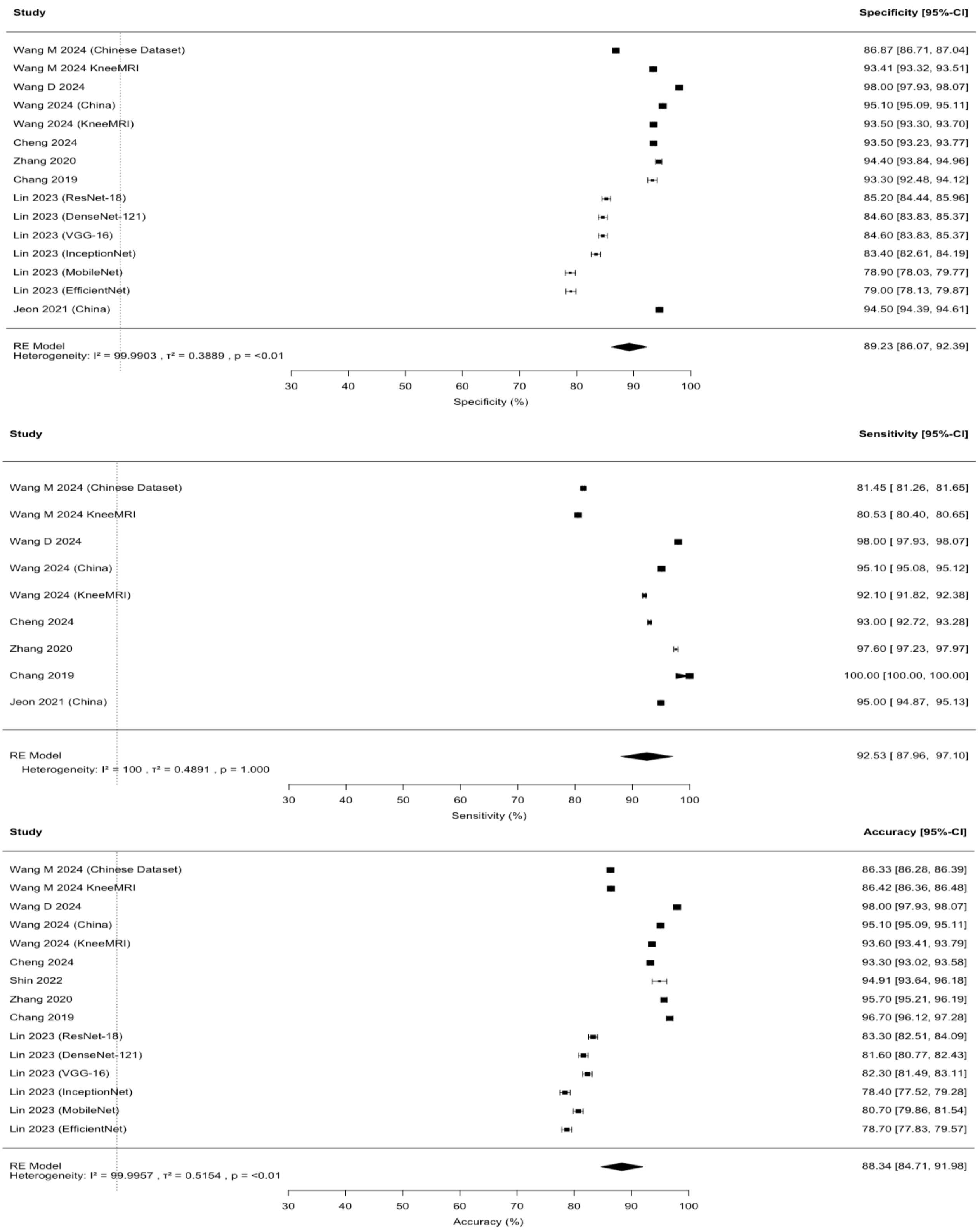

Figure s14: Forest Plot of *Outcomes of* Diagnosis of ACL Tears Using MRI Slice Depth of 3mm or more

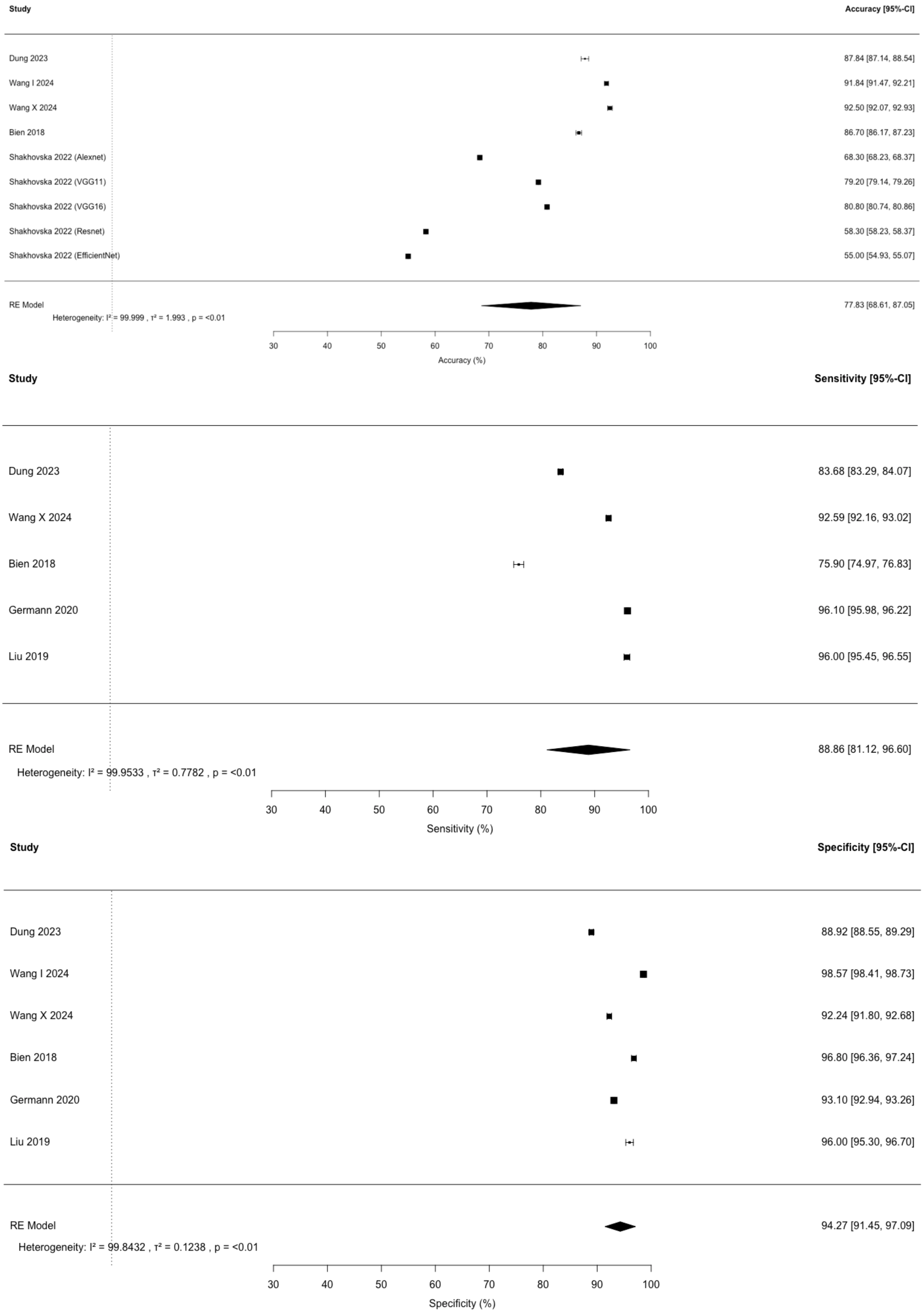

Figure s15: Forest Plot *of Outcomes of* Diagnosis of ACL Tears Using MRI Slice Depth of 2mm or more, up to 3mm

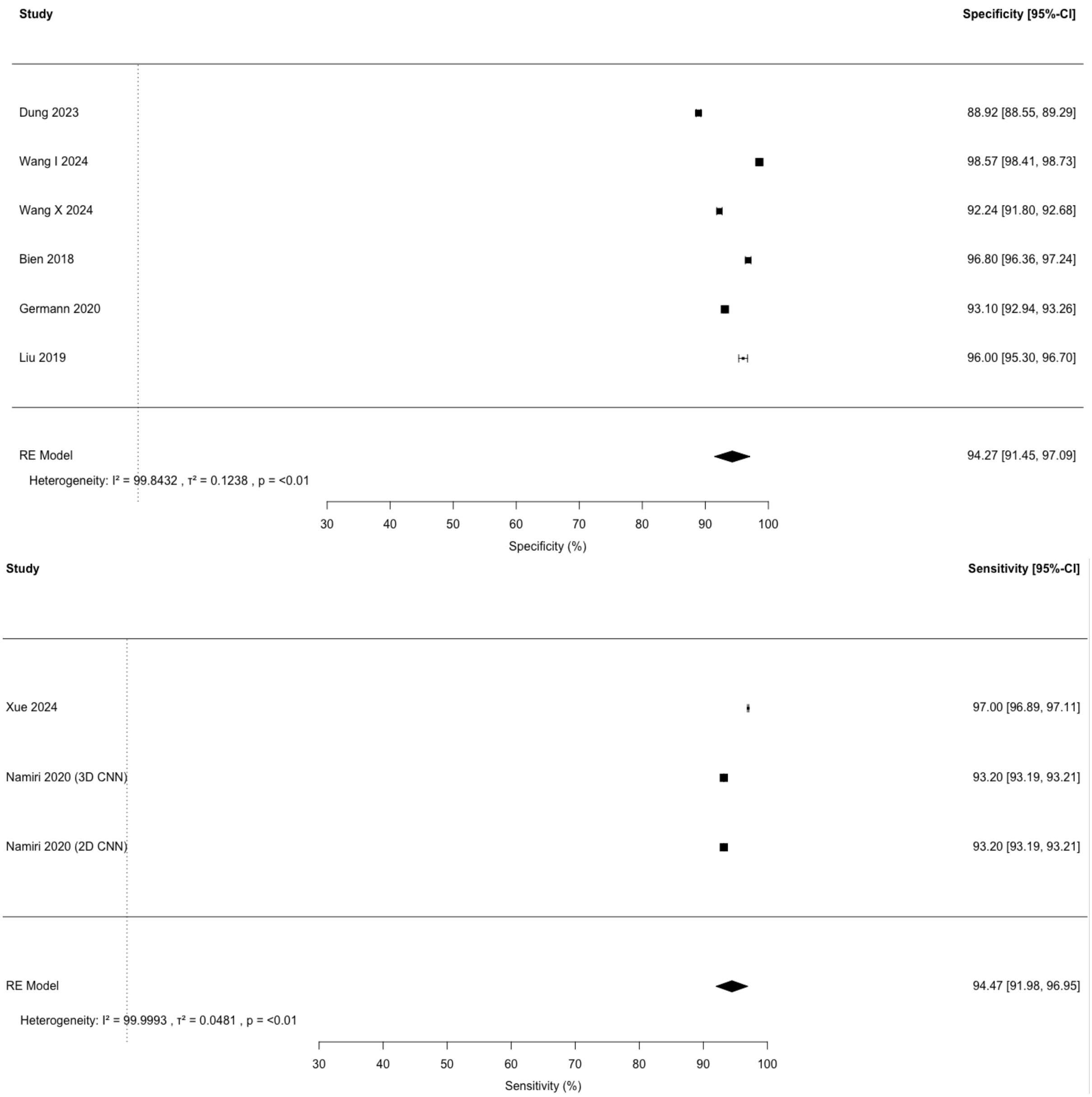

Figure s16: Forest Plot *of Outcomes of* Diagnosis of ACL Tears Using MRI Slice Depth of less than 2mm

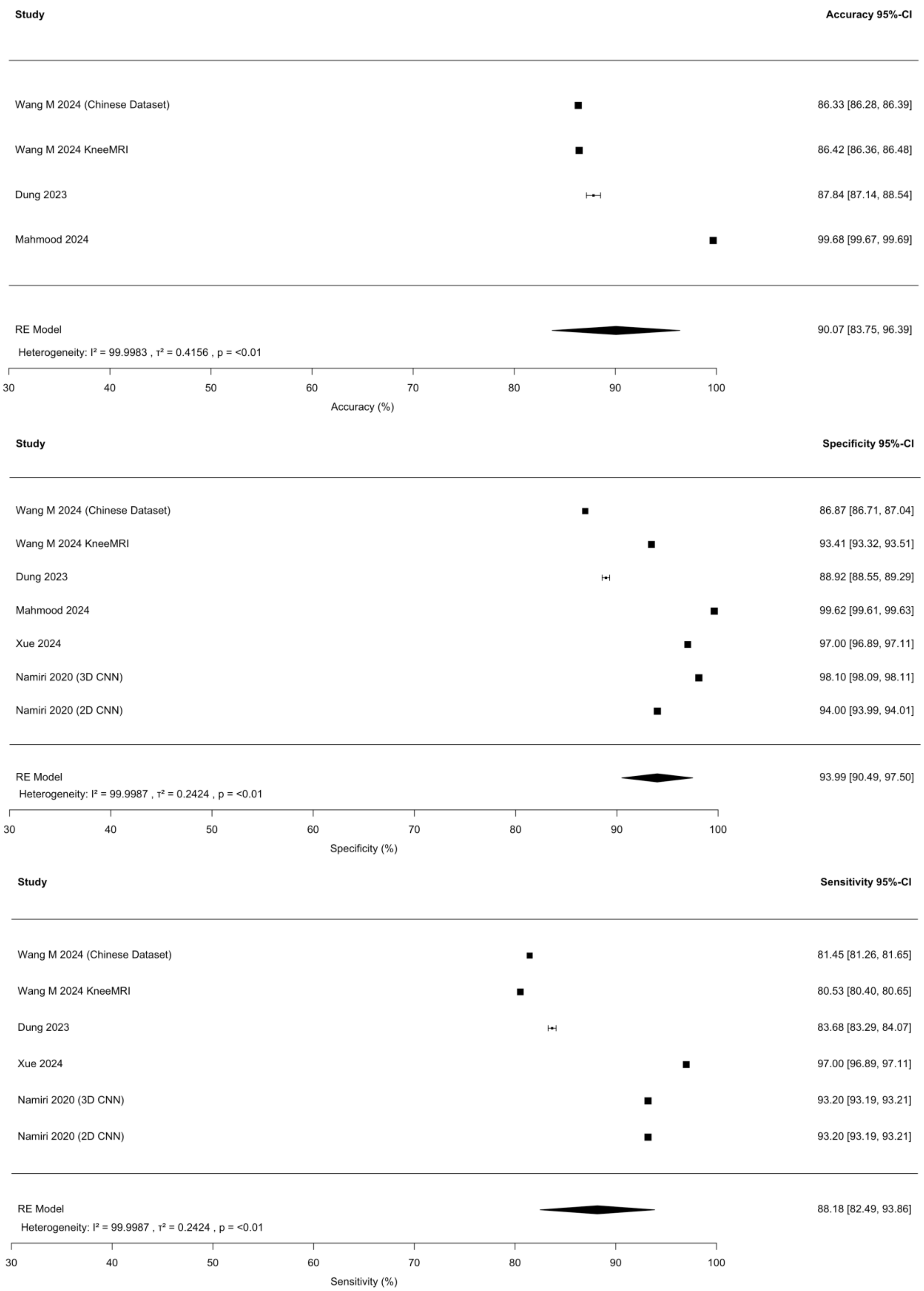

Figure s17: Forest Plot of Outcomes of Classification of MRI based on PD MRI sequence

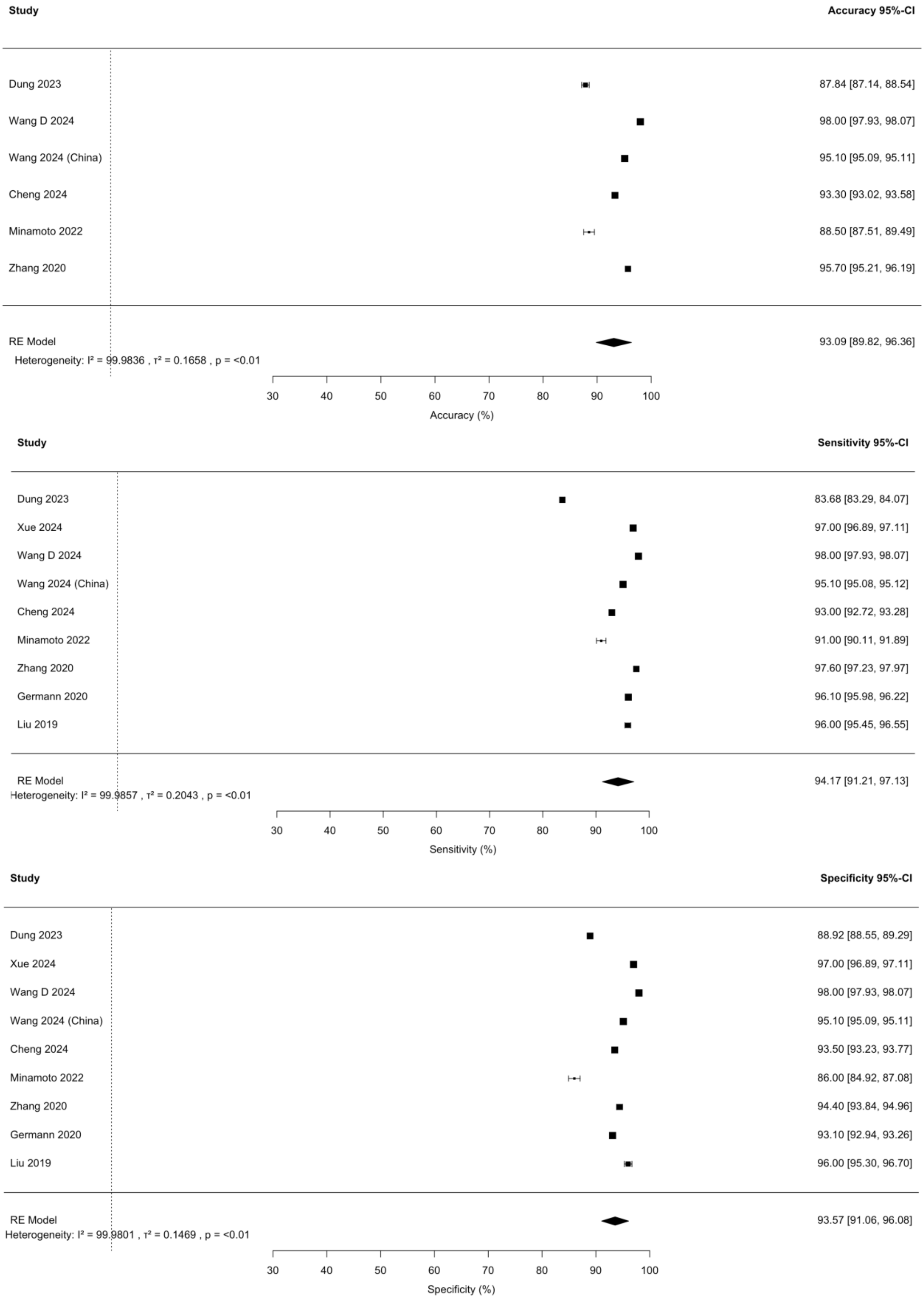

Figure s18: Forest Plot of Outcomes of Diagnosis of ACL tears using Arthroscopy as the Reference Standard

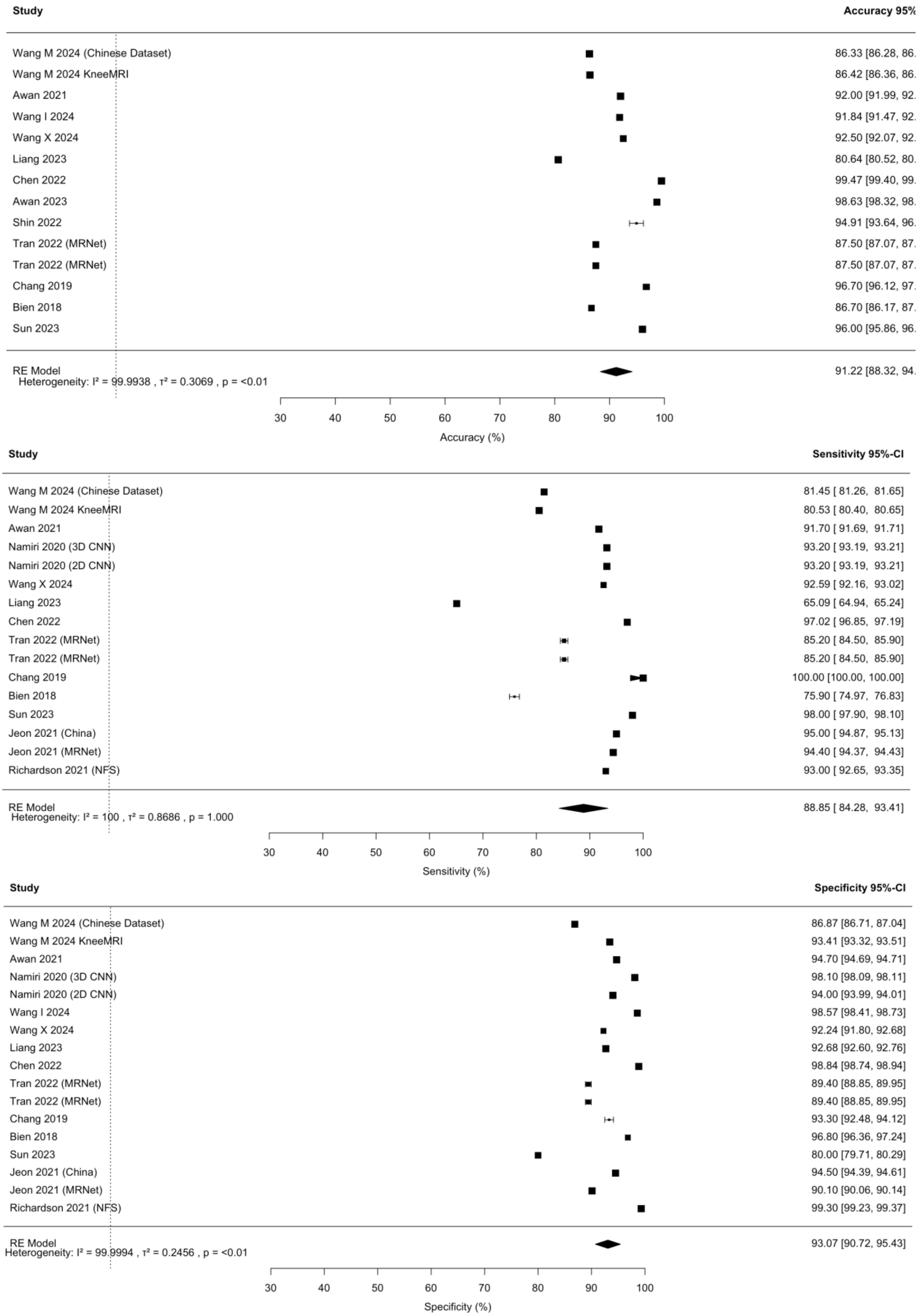

Figure s19: Forest Plot of Outcomes of Diagnosis of ACL tears using Clinician as the Reference Standard

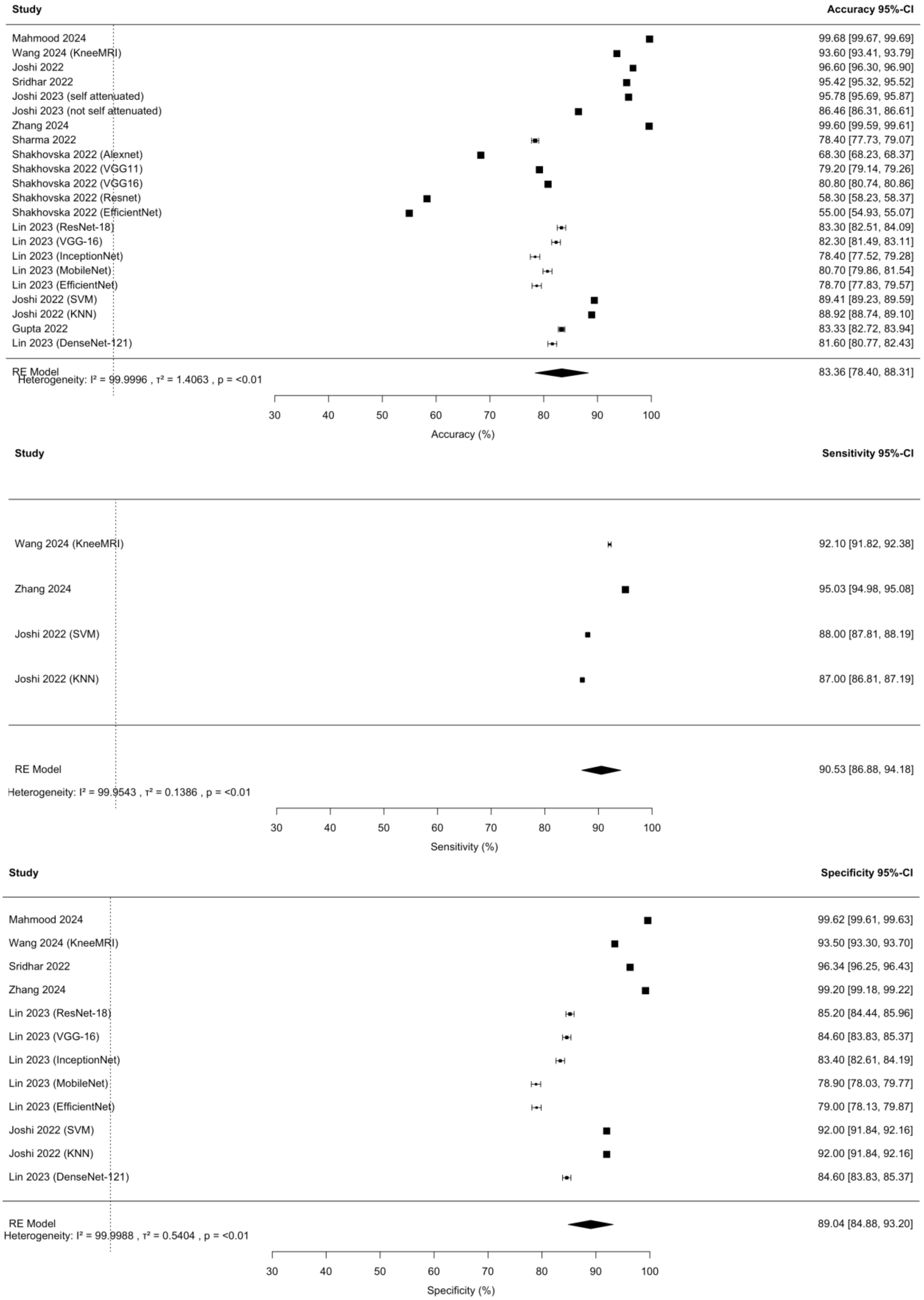

Figure s20: Forest Plot of Outcomes of Diagnosis of ACL tears using Dataset as the Reference Standard

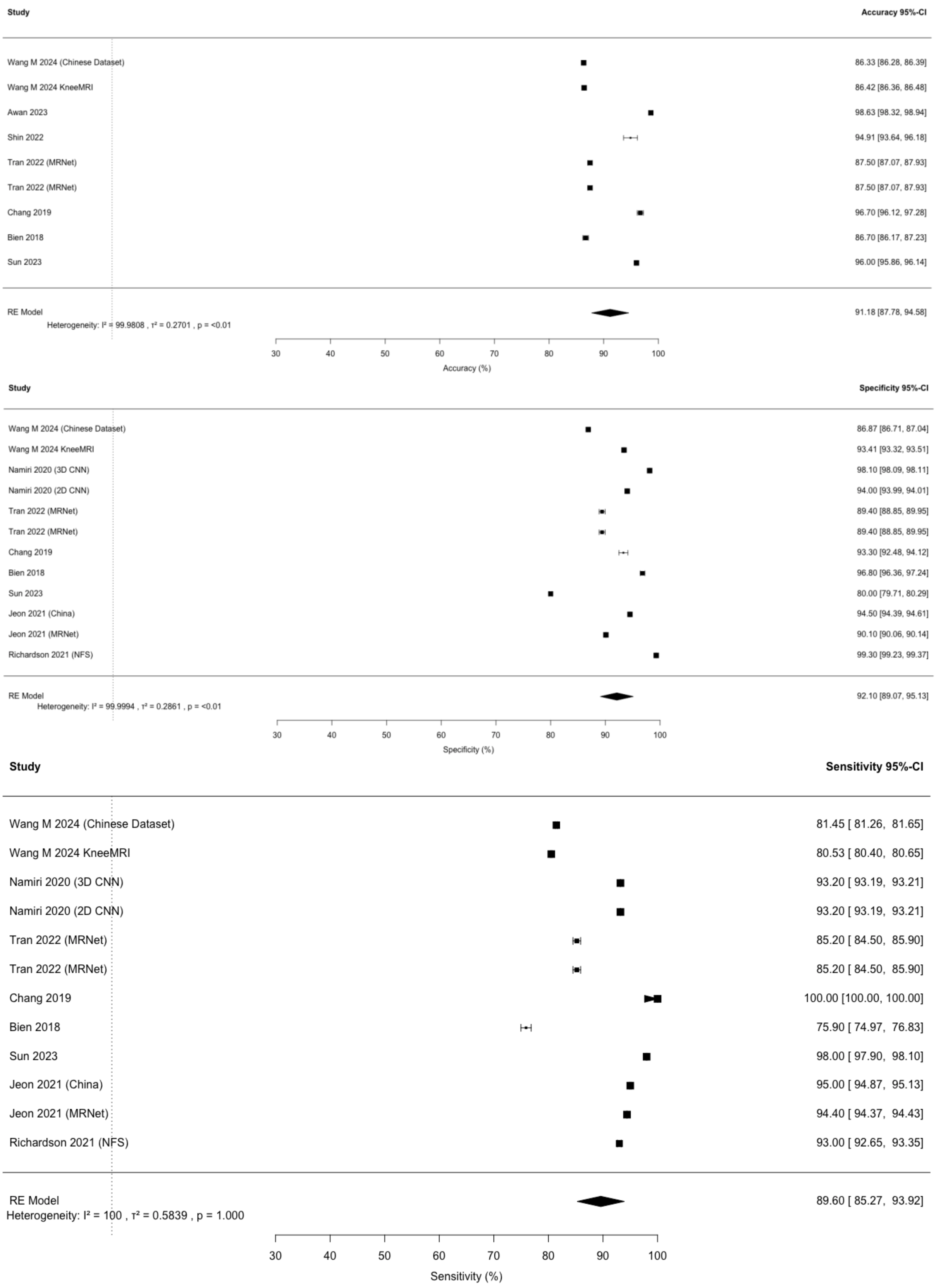

Figure s21: Forest Plot of Outcomes of Diagnosis of ACL tears using Radiologists as the Reference Standard

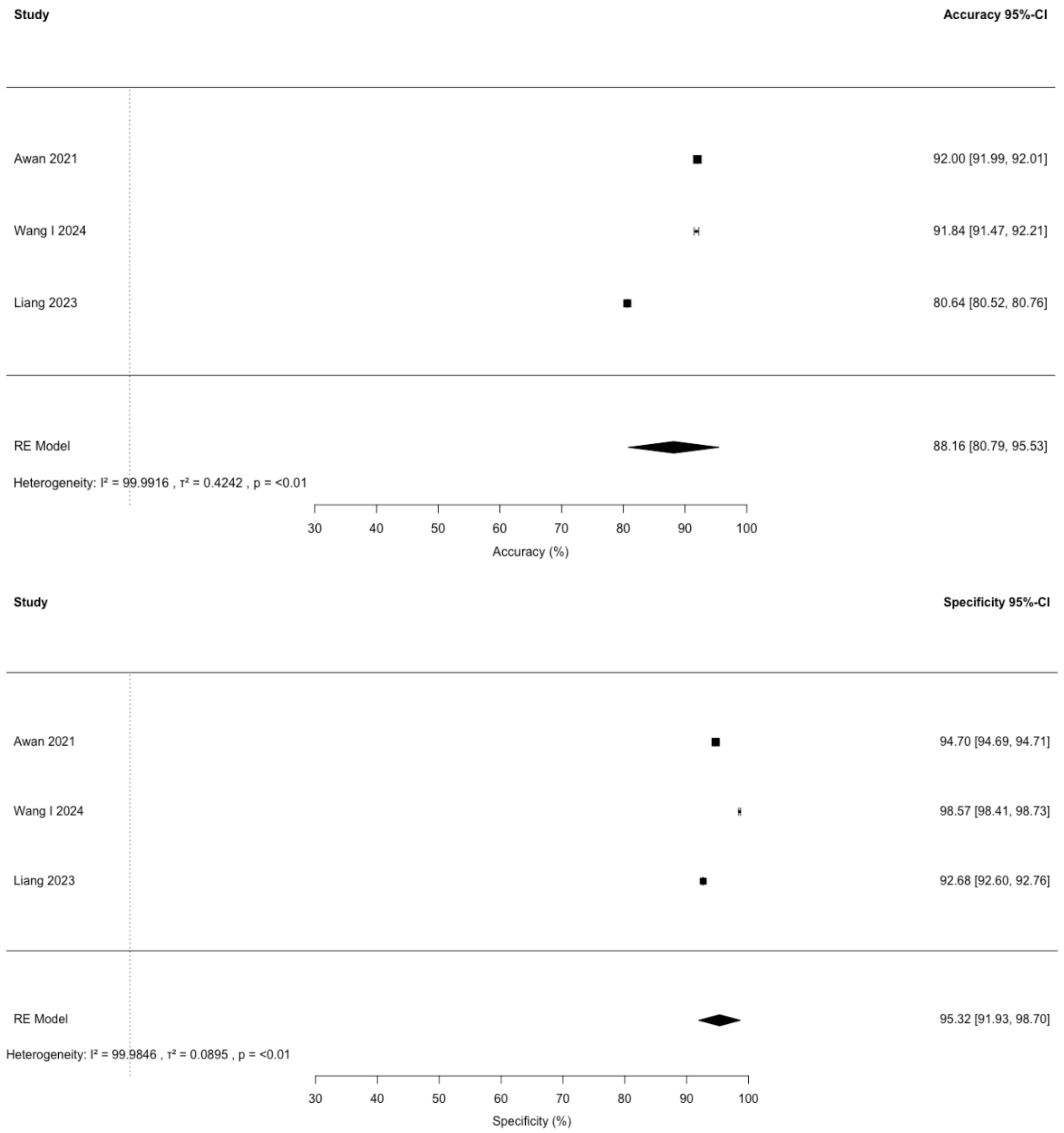

Figure s22: Forest Plot of Outcomes of Diagnosis of ACL tears using Orthopaedic Surgeons as the Reference Standard

Source	***Count***	***Search String***
Web of Science	*125*	*(TOPIC)*<br><br>*(AI or "artificial intelligence" or AIVI or "classification algorithm*" or "computer heuristic*" or "convolutional network*" or DALL-E or "decision support system*" or "decision tree" or DeepAI or "deep learning" or "data science" or "feature detection" or "generative pre-trained transformer" or "generative pretrained transformer" or Invideo or "language learning model*" or "large language model*" or "learning algorithm*" or "machine learning" or (Markov NEAR/3 model*) or Midjourney or ((multifactor* or multicriteria) NEAR/3 ("decision analysis" or "decision making")) or "natural language process*" or "nearest neighbo*" or "neural network*" or "outlier detection" or "pattern recognition" or Perplexity or "probability tree" or "random forest" or "representation learning" or Runway AI or Runway Gen-1 or "Stable Diffusion" or "support vector machine*" or "transfer learning" or "Bing chat" or ChatGPT* or "Chat GPT" or "Google* Bard" or "Google* Gemini" or "IBM Watson" or "Microsoft* Bing" or "Microsoft* Copilot" or OpenAI or "Open AI" or PathAI or "Path AI")*<br><br>*AND*<br><br>*Magnetic resonance or MRI or MRIs*<br><br>*AND*<br><br>*((anterior NEAr/2 cruciate* NEAR/2 ligament*) or acl)*
Scopus	*112*	*TITLE-ABS-KEY (("Artificial Intelligence" OR "AI" OR "Machine Learning" OR "Neural Networks" OR "Random Forest" OR "Artificial Neural Network" OR "ANN" OR "Support Vector Machine" OR "SVM" OR "Gradient Boosting" OR "Decision Trees" OR "Convolutional Neural Network" OR "CNN" OR "Computer Vision") AND ((acl OR “anterior cruciate ligament”) AND (injury OR tear)) AND knee AND (mri OR "Magnetic Resonance Imaging"))*
Embase	*105*	*1. (MRI or MRIs).tw,kw.*

		2. (MR adj3 (imag* or scan*)).tw,kw.<br>3. 1 or 2<br>4. “Knee*”.ab,dm,dv,fx,hw,kf,mf,ot,ti,tn,dq.<br>5. 3 and 4<br>6. Anterior cruciate ligament.ab,dm,dv,fx,hw,kf,mf,ot,ti,tn,dq.<br>7. Anterior cruciate ligament rupture.ab,dm,dv,fx,hw,kf,mf,ot,ti,tn,dq.<br>8. Anterior cruciate ligament injury.ab,dm,dv,fx,hw,kf,mf,ot,ti,tn,dq.<br>9. ((anterior adj2 cruciate* adj2 ligament*) or acl).tw.<br>10. Anterior cruciate ligament tear.ab,dm,dv,fx,hw,kf,mf,ot,ti,tn,dq.<br>11. 6 or 7 or 8 or 9 or 10<br>12. 5 and 11<br>13. exp artificial intelligence/ or exp deep learning/ or exp machine learning/ or (AI or “artificial intelligence” or AIVI or “classification algorithm*” or “computer heuristic*” or “convolutional network*” or DALL-E or “decision support system*” or “decision tree” or DeepAI or “deep learning” or “data science” or “feature detection” or “generative pre-trained transformer” or “generative pretrained transformer” or Invideo or “language learning model*” or “large language model*” or “learning algorithm*” or “machine learning” or (Markov adj3 model*) or Midjourney or ((multifactor* or multicriteria) adj3 (“decision analysis” or “decision making”)) or “natural language process*” or “nearest neighbo*” or “neural network*” or “outlier detection” or “pattern recognition” or Perplexity or “probability tree” or “random forest” or “representation learning” or Runway AI or Runway Gen-1 or “Stable Diffusion” or “support vector machine*” or “transfer learning” or “Bing chat” or ChatGPT* or “Chat GPT” or “Google* Bard” or “Google* Gemini” or “IBM Watson” or “Microsoft* Bing” or “Microsoft* Copilot” or OpenAI or “Open AI” or PathAI or “Path AI”).mp.<br>14. 12 and 13
MEDLINE	*86*	1. Magnetic Resonance Imaging/<br>2. (Magnetic resonance or MRI or MRIs).mp.<br>3. 1 or 2<br>4. Anterior Cruciate Ligament/<br>5. ((anterior adj2 cruciate* adj2 ligament*) or acl).mp.<br>6. 4 or 5

		7. *exp Artificial Intelligence/ or (AI or "artificial intelligence" or AIVI or "classification algorithm*" or "computer heuristic*" or "convolutional network*" or DALL-E or "decision support system*" or "decision tree" or DeepAI or "deep learning" or "data science" or "feature detection" or "generative pre-trained transformer" or "generative pretrained transformer" or Invideo or "language learning model*" or "large language model*" or "learning algorithm*" or "machine learning" or (Markov adj3 model*) or Midjourney or ((multifactor* or multicriteria) adj3 ("decision analysis" or "decision making")) or "natural language process*" or "nearest neighbo*" or "neural network*" or "outlier detection" or "pattern recognition" or Perplexity or "probability tree" or "random forest" or "representation learning" or Runway AI or Runway Gen-1 or "Stable Diffusion" or "support vector machine*" or "transfer learning" or "Bing chat" or ChatGPT* or "Chat GPT" or "Google* Bard" or "Google* Gemini" or "IBM Watson" or "Microsoft* Bing" or "Microsoft* Copilot" or OpenAI or "Open AI" or PathAI or "Path AI").mp.*<br>8. *3 and 6 and 7*
PubMed	*85*	*Knee AND (ACL OR "Anterior Cruciate Ligament") AND (Injur* OR Injury OR Injuries OR Tear OR Rupture) AND ("Artificial Intelligence" OR "AI" OR "Machine Learning" OR "Neural Networks" OR "Random Forest" OR "Artificial Neural Network" OR "ANN" OR "Support Vector Machine" OR "SVM" OR "Gradient Boosting" OR "Decision Trees" OR "Convolutional Neural Network" OR "CNN" OR "Computer Vision") AND (Diagnosis OR Prognosis OR Prognostication OR Prognosis OR Identification OR Identify OR Identif* OR Detection) AND (MRI OR "Magnetic Resonance Imaging")*

Table s1 –Search strategy

Inclusion	*Exclusion*
Studies relating to the use of AI to detect or classify ACL tears on MRI	*Studies not focussing on MRI*
Studies with data pertaining to at least one of the: sensitivity, specificity or AUC of the proposed model	*Studies not focussed on ACL tears*
Automated or semi-automated AI	*Studies not using a deep learning (AI) based detection algorithm*
Retrospective studies	*Reviews, case studies, editorials, commentaries, conference abstracts and theses*
Peer reviewed studies, published in a scientific journal	*Studies that are not in English*
	Full text not available

Table s2: Inclusion criteria

Extraction categories
Year of publication
First author
Country of origin
Title
Sample size
Type of AI used
Ground truth
Domain
Radiomics model used
Outcome measures defining model performance
Comparison to clinicians, and its subsequent outcome measures defining performance
Type of MRI

Table s3: Extracted data

Domain	*Outcome*
Population	*Patients undergoing knee MRI with suspected anterior cruciate ligament (ACL) injury.*
Intervention	*Artificial intelligence (AI) models used to detect or classify ACL tears on MRI.*
Comparator	*Human clinicians (radiologists or orthopaedic surgeons), or other AI models, where available.*
Outcome	*Diagnostic performance measures including sensitivity, specificity and accuracy, with secondary outcomes including F1 score, Dice coefficient, area under the curve (AUC), and comparison to clinical decision-making where reported.*

Table s4: PICO

Study	Title	Domain	N	Clinician Assistance/ Comparison	Ground Truth	Type of Clinician	Accuracy (%)	Sensitivity (%)	Specificity (%)	Other Metrics
Chen 2023	*A transfer learning approach for staging diagnosis of anterior cruciate ligament injury on a new modified MR dual precision positioning of thin-slice oblique sagittal FS-PDWI sequence.*	*Classification*	*6*	*Comparison*	*Arthroscopy*	*Consensus of 2 radiologists*	*87.8 (Clinician); 95.3 (AI)*	*87.87 (Clinician); 95.31 (AI)*	*95.54 (Clinician); 98.4 (AI)*	*-*
Xue 2024	*Approaching expert-level accuracy for differentiating ACL tear types on MRI with deep learning.*	*Classification*	*862*	*Comparison*	*Arthroscopy*	*junior and senior radiologist and orthopaedic surgeon*	*93.67 (Clinician); 94.0 (AI)*	*-*	*-*	*AUC: 0.92 (Clinician); 0.99 (AI)*
Wang J 2024	*Lightweight Attentive Graph Neural Network with Conditional*	*Diagnosis*	*147*	*Comparison*	*Independent Orthopaedic surgeons*	*6 orthopaedic surgeons: 2 juniors, 2*	*80.5 (Clinician); 91.84 (AI)*	*-*	*95.89 (Clinician); 98.57 (AI)*	*F1: 82.6 (Clinician); 92.35 (AI); Precision: 86.46 (Clinician); 93.66 (AI)*

	Random Field for Diagnosis of Anterior Cruciate Ligament Tear.					*intermediate, 2 seniors*				
Chen 2022	*Artificial Intelligence-Assisted Diagnosis of Anterior Cruciate Ligament Tears From Magnetic Resonance Images: Algorithm Development and Validation Study.*	*Diagnosis*	*40*	*Comparison*	*Independent Radiologist and Orthopaedic Surgeon*	*Group 1 includes senior trainees (chief residents and sports fellows), Group 2 mid-level trainees (third- and fourth-year residents), and Group 3 junior trainees (first- and second-year residents). 3 in each group, all of which orthopaedi*	*81.6 (Clinician); 97.5 (AI)*	*-*	*-*	*-*

						c surgeons.				
Minamoto 2022	*Automated detection of anterior cruciate ligament tears using a deep convolutional neural network.*	*Diagnosis*	*200*	*Comparison*	*Arthroscopy*	*10 knee surgeons and 2 radiologists*	*84.25 (Clinician); 88.5 (AI)*	*93.33 (Clinician); 91.0 (AI)*	*76.58 (Clinician); 86.0 (AI)*	*-*
Zhang 2020	*Deep Learning Approach for Anterior Cruciate Ligament Lesion Detection: Evaluation of Diagnostic Performance Using Arthroscopy as the Reference Standard.*	*Diagnosis*	*81*	*Comparison*	*Arthroscopy*	*3 senior radiologists and 3 inexperienced radiologists*	*85.7 (Clinician); 95.7 (AI)*	*88.1 (Clinician); 97.6 (AI)*	*84.0 (Clinician); 94.4 (AI)*	*-*
Bien 2018	*Deep-learning-assisted diagnosis for*	*Diagnosis*	*120*	*Comparison*	*Independent Radiologists*	*MSK radiologists*	*92.0 (Clinician); 86.7 (AI)*	*90.6 (Clinician); 75.9 (AI)*	*93.3 (Clinician); 96.8 (AI)*	*-*

	knee magnetic resonance imaging: Development and retrospective validation of MRNet.									
Germann 2020	*Deep Convolutional Neural Network-Based Diagnosis of Anterior Cruciate Ligament Tears: Performance Comparison of Homogenous Versus Heterogeneous Knee MRI Cohorts With Different Pulse Sequence Protocols and 1.5-T and 3-T Magnetic Field Strengths.*	*Diagnosis*	*512*	*Comparison*	*Arthroscopy*	*3 radiologists*	*-*	*97.77 (Clinician); 96.1 (AI)*	*99.87 (Clinician); 93.1 (AI)*	*AUC: 0.99 (Clinician); 0.935 (AI)*

Liu 2019	*Fully Automated Diagnosis of Anterior Cruciate Ligament Tears on Knee MR Images by Using Deep Learning.*	*Diagnosis*	*100*	*Comparison*	*Arthroscopy*	*MSK radiologist, MSK radiologist fellow, 3 radiology residents*	*-*	*96.0 (Clinician); 96.0 (AI)*	*96.0 (Clinician); 96.0 (AI)*	*AUC: 0.98 (Clinician); 0.98 (AI)*
Astuto 2021	*Automatic Deep Learning-assisted Detection and Grading of Abnormalities in Knee MRI Studies.*	*Classification*	*250*	*Assistance*	*Independent Radiologist*	*2 trainees and 2 attending radiologists*	*-*	*85.63 (Clinician); 86.6 (AI)*	*-*	*-*
Wang 2024	*A Deep Learning Model Enhances Clinicians' Diagnostic Accuracy to More Than 96% for Anterior Cruciate Ligament Ruptures on*	*Diagnosis*	*110*	*Assistance*	*Knee MRI Dataset*	*38 clinicians, 9 sports med experts, 15 sports med trainees, 6 radiology experts, 8 radiology trainees*	*88.97 (Clinician); 96.67 (AI)*	*90.13 (Clinician); 93.94 (AI)*	*88.37 (Clinician); 98.01 (AI)*	*-*

	Magnetic Resonance Imaging.									
Wang M 2024 (OAI)	*One-stop detection of anterior cruciate ligament injuries on magnetic resonance imaging using deep learning with multicenter validation.*	*Classification*	*17*	*Comparison*	*OAI Dataset*	*2 Experienced radiologists*	*87.1 (Clinician); 96.8 (AI)*	*82.3 (Clinician); 100 (AI)*	*92.2 (Clinician); 95.9 (AI)*	*-*
Wang M 2024 (Chinese)	*One-stop detection of anterior cruciate ligament injuries on magnetic resonance imaging using deep learning with multicenter validation.*	*Classification*	*170*	*Comparison*	*Arthroscopy*	*2 Experienced radiologists*	*84.5 (Clinician); 89.1 (AI)*	*83.7 (Clinician); 77.3 (AI)*	*86.4 (Clinician); 89.1 (AI)*	*-*

Table s5 – Clinician comparison

The table compares diagnostic performance across studies evaluating clinician and AI-assisted diagnostics. Each entry lists the study, domain (classification or diagnosis), sample size, and the role of AI (either clinician assistance or direct comparison with clinicians). Clinician expertise is specified, with accuracy, sensitivity, and specificity metrics included. Additional metrics (e.g., AUC, F1 Score, Precision) are consolidated under "Other Metrics."

Study	Title	Validation method	Imaging plane	Scan sequence	Scanning thickness	Strength (Tesla)
Wang M 2024 (Chinese Dataset)	One-stop detection of anterior cruciate ligament injuries on magnetic resonance imaging using deep learning with multicenter validation.	External validation dataset post-retraining	Sagittal	Proton Density-Weighted Spectral Attenuated Inversion Recovery (PDW-SPAIR)	3.5 mm	1.5T (Achieva) and 3T (Ingenia)
Wang M 2024 (KneeMRI)	One-stop detection of anterior cruciate ligament injuries on magnetic resonance imaging using deep learning with multicenter validation.	External validation dataset post-retraining	Sagittal	T1-weighted, T2 with fat saturation, PD-weighted, T2 with fat saturation, and PD-weighted with fat saturation	3.5 mm	1.5T (Siemens Avanto MRI scanner)
Awan 2021	Efficient Detection of Knee Anterior Cruciate Ligament from Magnetic Resonance Imaging Using Deep Learning Approach.	5-Fold Cross-Validation (17 MRIs per validation fold).	Sagittal, Coronal	PDW with T2-weighted Turbo Spin-Echo (Sagittal), T1-weighted Turbo Spin-Echo (Coronal) and FS	2.5 to 3.3 mm	3.0 T (Philips Achieva)
Dung 2023	End-to-end deep learning model for segmentation and severity staging of anterior cruciate ligament injuries from MRI.	Semi-supervised validation with pseudo labeling (training: 247 cases, testing: 50 cases)	Sagittal	Proton Density (PD) Fast Spin-Echo (FSE) with FS	2.5 mm	1.5 T (MAGNETOM Skyra, Siemens)
Mahmood 2024	Acute Knee Injury Detection with Magnetic Resonance Imaging (MRI)	Random split: Training (70%), Validation (15%), Testing (15%)	Sagittal, Coronal	Proton Density-Weighted Fat Suppression (FS-PDWI), T1 and T2 weighted	Not specified	1.5 T (Siemens Avanto MRI)
Chen 2023	A transfer learning approach for staging diagnosis of anterior cruciate ligament injury on a	Random split: Training (70%),	Sagittal oblique	Dual Precision Positioning Thin-Slice Oblique Sagittal FS-	2.3 mm	1.5 T (Magnetom Espree, Siemens)

	new modified MR dual precision positioning of thin-slice oblique sagittal FS-PDWI sequence.	*Validation (10%), Testing (20%)*		*PDWI (DPP-TSO-Sag-FS-PDWI)*		
Xue 2024	*Approaching expert-level accuracy for differentiating ACL tear types on MRI with deep learning.*	*Random split: Training (80%), Validation (10%), Testing (10%)*	*Sagittal*	*Proton Density-Weighted Spectral Attenuated Inversion Recovery (PDW-SPAIR)*	*1.5 mm*	*3.0 T (Philips Ingenia)*
Namiri 2020 (3D CNN)	*Deep Learning for Hierarchical Severity Staging of Anterior Cruciate Ligament Injuries from MRI.*	*Random split: Training (70%), Validation (10%), Testing (20%)*	*Sagittal oblique*	*Proton Density-Weighted 3D Fast Spin-Echo (CUBE)*	*0.5 mm*	*3.0 T (GE Healthcare MRI Scanners)*
Namiri 2020 (2D CNN)	*Deep Learning for Hierarchical Severity Staging of Anterior Cruciate Ligament Injuries from MRI.*	*Random split: Training (70%), Validation (10%), Testing (20%)*	*Sagittal oblique*	*Proton Density-Weighted 3D Fast Spin-Echo (CUBE)*	*0.5 mm*	*3.0 T (GE Healthcare MRI Scanners)*
Wang J 2024	*Lightweight Attentive Graph Neural Network with Conditional Random Field for Diagnosis of Anterior Cruciate Ligament Tear.*	*Random split: Training (70%), Validation (10%), Testing (20%)*	*Sagittal and Coronal Oblique*	*Not specified*	*Not specified*	*1.5 T and 3.0 T*

Wang X 2024	*Deep Learning-Assisted Automatic Diagnosis of Anterior Cruciate Ligament Tear in Knee Magnetic Resonance Images.*	*5-fold Cross-Validation*	*Sagittal, Coronal, Axial*	*Proton Density-Weighted Imaging, T2weighted with FS*	*2.5 mm*	*1.5 T and 3.0 T*
Wang D 2024	*Improving inceptionV4 model based on fractional-order snow leopard optimization algorithm for diagnosing of ACL tears.*	*Random split: Training (70%), Testing (30%)*	*Sagittal and Coronal*	*Proton Density-Weighted Imaging with Fat Suppression (FS-PDWI)*	*3.0 mm*	*1.5 T and 3.0 T*
Wang 2024 (China)	*A Deep Learning Model Enhances Clinicians' Diagnostic Accuracy to More Than 96% for Anterior Cruciate Ligament Ruptures on Magnetic Resonance Imaging.*	*Random split (Training: 80%, Validation: 10%, Testing: 10%)*	*Sagittal*	*T1-Weighted, T2-weighted Proton Density-Weighted Imaging*	*3.0 mm*	*1.5 T and 3.0 T*
Wang 2024 (KneeMRI)	*A Deep Learning Model Enhances Clinicians' Diagnostic Accuracy to More Than 96% for Anterior Cruciate Ligament Ruptures on Magnetic Resonance Imaging.*	*External validation only*	*Sagittal*	*T1-Weighted, T2-weighted Proton Density-Weighted Imaging with FS*	*3.5 mm*	*1.5 T (Siemens Avanto)*
Cheng 2024	*Application of machine learning-based multi-sequence MRI radiomics in diagnosing anterior cruciate ligament tears.*	*Random split: Training (80%), Validation (20%)*	*Sagittal*	*T1-weighted imaging and Proton Density-Weighted Imaging with Fat Suppression*	*4.0 mm*	*1.5 T (Siemens Avanto MRI)*
Liang 2023	*Effective automatic detection of anterior cruciate ligament injury using convolutional neural network with two attention mechanism modules.*	*Fivefold cross-validation*	*Sagittal*	*T1-weighted imaging*	*Not specified*	*Not specified*

Chen 2022	*Artificial Intelligence-Assisted Diagnosis of Anterior Cruciate Ligament Tears From Magnetic Resonance Images: Algorithm Development and Validation Study.*	*Random split: Training (80%), Testing (20%)*	*Sagittal*	*Proton Density-Weighted Imaging*	*Not specified*	*Various MRI scanners (1.5 T and 3.0 T)*
Awan 2023	*MGACA-Net: a novel deep learning based multi-scale guided attention and context aggregation for localization of knee anterior cruciate ligament tears region in MRI images.*	*Random split: Training (75%), Validation (25%)*	*Sagittal, Coronal*	*Proton Density-Weighted Imaging with FS*	*Not specified*	*1.5 T and 3.0 T*
Shin 2022	*Development of convolutional neural network model for diagnosing tear of anterior cruciate ligament using only one knee magnetic resonance image.*	*Random split: Training (79%), Testing (21%)*	*Oblique-Sagittal*	*Fat-suppressed T2-weighted imaging*	*4 mm*	*1.5 T (Philips)*
Tran 2022 (MRNet)	*Deep learning to detect anterior cruciate ligament tear on knee MRI: multi-continental external validation.*	*Random split: Training (70%), Validation (20%), Testing (10%)*	*Coronal and Sagittal*	*Proton Density-Weighted and T2-Weighted with Fat Suppression*	*Not specified*	*1.0 T, 1.5 T, 3.0 T*
Tran 2022 (MRNet)	*Deep learning to detect anterior cruciate ligament tear on knee MRI: multi-continental external validation.*	*Random split: Training (70%), Validation (20%), Testing (10%)*	*Coronal, Sagittal, Axial*	*Proton Density-Weighted, T2-Weighted Imaging with Fat Suppression*	*Not specified*	*1.0 T, 1.5 T, 3.0 T*

Minamoto 2022	*Automated detection of anterior cruciate ligament tears using a deep convolutional neural network.*	*5-fold Cross-Validation*	*Sagittal*	*Proton Density-Weighted Imaging*	*0.7–3.0 mm*	*1.5 T and 3.0 T*
Sridhar 2022	*A Torn ACL Mapping in Knee MRI Images Using Deep Convolution Neural Network with Inception-v3.*	*Random split: Training (70%), Testing (30%)*	*Coronal, Sagittal, Axial*	*Proton Density-Weighted, T2-Weighted Imaging with Fat Suppression*	*Not specified*	*1.5 T and 3.0 T*
Zhang 2020	*Deep Learning Approach for Anterior Cruciate Ligament Lesion Detection: Evaluation of Diagnostic Performance Using Arthroscopy as the Reference Standard.*	*5-fold Cross-Validation*	*Sagittal*	*Proton Density-Weighted Spectral Attenuated Inversion Recovery (PDW-SPAIR)*	*3.5 mm*	*1.5 T and 3.0 T*
Chang 2019	*Deep Learning for Detection of Complete Anterior Cruciate Ligament Tear.*	*5-fold Cross-Validation*	*Coronal*	*Proton Density (PD) Non-Fat-Suppressed*	*3.0 mm*	*1.5 T and 3.0 T*
Bien 2018	*Deep-learning-assisted diagnosis for knee magnetic resonance imaging: Development and retrospective validation of MRNet.*	*5-fold Cross-Validation*	*Sagittal, Coronal, Axial*	*coronal T1 weighted, coronal T2 with fat saturation, sagittal proton density (PD) weighted, sagittal T2 with fat saturation, and axial PD weighted with fat saturation*	*2.0–3.0 mm*	*1.5 T and 3.0 T*
Joshi 2023 (self attenuated)	*Anterior cruciate ligament tear detection based on convolutional neural network and generative adversarial neural network*	*Random split: Training (70%), Testing (30%)*	*Coronal, Sagittal, Axial*	*Proton Density-Weighted, T2-Weighted Imaging with GAN-Augmented Data*	*Not specified*	*1.5 T and 3.0 T*

Joshi 2023 (not self attenuated)	*Anterior cruciate ligament tear detection based on convolutional neural network and generative adversarial neural network*	*Random split: Training (70%), Testing (30%)*	*Coronal, Sagittal, Axial*	*Proton Density-Weighted, T2-Weighted Imaging with GAN-Augmented Data*	*Not specified*	*1.5 T and 3.0 T*
Sun 2023	*Anterior cruciate ligament tear detection based on deep belief networks and improved honey badger algorithm*	*Random split: Training (70%), Testing (30%)*	*Sagittal, Coronal*	*Proton Density-Weighted Imaging FS*	*Not specified*	*1.5 T and 3.0 T*
Zhang 2024	*A new optimization method for accurate anterior cruciate ligament tear diagnosis using convolutional neural network and modified golden search algorithm*	*Train/Test split: 75%/25%*	*Sagittal, Coronal, Axial*	*coronal T1 weighted, coronal T2 with fat saturation, sagittal proton density (PD) weighted, sagittal T2 with fat saturation, and axial PD weighted with fat saturation*	*Not specified*	*1.5 T and 3.0 T*
Sharma 2022	*A ResNet50-Based Approach to Detect Multiple Types of Knee Tears Using MRIs*	*Random split: Training (82.5%), Validation (8.75%), Testing (8.75%)*	*Sagittal, Coronal, Axial*	*Proton Density-Weighted and T2-Weighted Imaging*	*Not specified*	*1.5 T and 3.0 T*
Shakhovska 2022 (Alexnet)	*Comparative Analysis of Backbone Networks for Deep Knee MRI Classification Models*	*5-fold Cross-Validation*	*Sagittal, Coronal, Axial*	*coronal T1 weighted, coronal T2 with fat saturation, sagittal proton density (PD) weighted, sagittal T2 with fat saturation, and*	*2.0–3.0 mm*	*1.5 T and 3.0 T*

				axial PD weighted with fat saturation		
Shakhovska 2022 (VGG11)	*Comparative Analysis of Backbone Networks for Deep Knee MRI Classification Models*	*5-fold Cross-Validation*	*Sagittal, Coronal, Axial*	*coronal T1 weighted, coronal T2 with fat saturation, sagittal proton density (PD) weighted, sagittal T2 with fat saturation, and axial PD weighted with fat saturation*	*2.0–3.0 mm*	*1.5 T and 3.0 T*
Shakhovska 2022 (VGG16)	*Comparative Analysis of Backbone Networks for Deep Knee MRI Classification Models*	*5-fold Cross-Validation*	*Sagittal, Coronal, Axial*	*coronal T1 weighted, coronal T2 with fat saturation, sagittal proton density (PD) weighted, sagittal T2 with fat saturation, and axial PD weighted with fat saturation*	*2.0–3.0 mm*	*1.5 T and 3.0 T*
Shakhovska 2022 (Resnet)	*Comparative Analysis of Backbone Networks for Deep Knee MRI Classification Models*	*5-fold Cross-Validation*	*Sagittal, Coronal, Axial*	*coronal T1 weighted, coronal T2 with fat saturation, sagittal proton density (PD) weighted, sagittal T2 with fat saturation, and axial PD weighted with fat saturation*	*2.0–3.0 mm*	*1.5 T and 3.0 T*
Shakhovska 2022 (EfficientNet)	*Comparative Analysis of Backbone Networks for Deep Knee MRI Classification Models*	*5-fold Cross-Validation*	*Sagittal, Coronal, Axial*	*coronal T1 weighted, coronal T2 with fat saturation, sagittal proton density (PD) weighted, sagittal T2 with fat saturation, and*	*2.0–3.0 mm*	*1.5 T and 3.0 T*

				axial PD weighted with fat saturation		
Lin 2023 (ResNet-18)	*A Channel Correction and Spatial Attention Framework for Anterior Cruciate Ligament Tear with Ordinal Loss*	*5-fold Cross-Validation*	*Sagittal, Coronal, Axial*	*coronal T1 weighted, coronal T2 with fat saturation, sagittal proton density (PD) weighted, sagittal T2 with fat saturation, and axial PD weighted with fat saturation*	*3.0 mm*	*1.5 T and 3.0 T*
Lin 2023 (DenseNet-121)	*A Channel Correction and Spatial Attention Framework for Anterior Cruciate Ligament Tear with Ordinal Loss*	*5-fold Cross-Validation*	*Sagittal, Coronal, Axial*	*coronal T1 weighted, coronal T2 with fat saturation, sagittal proton density (PD) weighted, sagittal T2 with fat saturation, and axial PD weighted with fat saturation*	*3.0 mm*	*1.5 T and 3.0 T*
Lin 2023 (VGG-16)	*A Channel Correction and Spatial Attention Framework for Anterior Cruciate Ligament Tear with Ordinal Loss*	*5-fold Cross-Validation*	*Sagittal, Coronal, Axial*	*coronal T1 weighted, coronal T2 with fat saturation, sagittal proton density (PD) weighted, sagittal T2 with fat saturation, and axial PD weighted with fat saturation*	*3.0 mm*	*1.5 T and 3.0 T*
Lin 2023 (InceptionNet)	*A Channel Correction and Spatial Attention Framework for Anterior Cruciate Ligament Tear with Ordinal Loss*	*5-fold Cross-Validation*	*Sagittal, Coronal, Axial*	*coronal T1 weighted, coronal T2 with fat saturation, sagittal proton density (PD) weighted, sagittal T2 with fat saturation, and*	*3.0 mm*	*1.5 T and 3.0 T*

				axial PD weighted with fat saturation		
Lin 2023 (MobileNet)	*A Channel Correction and Spatial Attention Framework for Anterior Cruciate Ligament Tear with Ordinal Loss*	*5-fold Cross-Validation*	*Sagittal, Coronal, Axial*	*coronal T1 weighted, coronal T2 with fat saturation, sagittal proton density (PD) weighted, sagittal T2 with fat saturation, and axial PD weighted with fat saturation*	*3.0 mm*	*1.5 T and 3.0 T*
Lin 2023 (EfficientNet)	*A Channel Correction and Spatial Attention Framework for Anterior Cruciate Ligament Tear with Ordinal Loss*	*5-fold Cross-Validation*	*Sagittal, Coronal, Axial*	*coronal T1 weighted, coronal T2 with fat saturation, sagittal proton density (PD) weighted, sagittal T2 with fat saturation, and axial PD weighted with fat saturation*	*3.0 mm*	*1.5 T and 3.0 T*
Joshi 2022 (SVM)	*Anterior Cruciate Ligament Tear Detection In Mri Images Using Multi-Neighbor Local Binary Pattern*	*Random split: Training (70%), Testing (30%)*	*Sagittal, Coronal, Axial*	*Proton Density-Weighted and T1, T2-Weighted Imaging*	*Not specified*	*1.5 T and 3.0 T*
Joshi 2022 (KNN)	*Anterior Cruciate Ligament Tear Detection In Mri Images Using Multi-Neighbor Local Binary Pattern*	*Random split: Training (70%), Testing (30%)*	*Sagittal, Coronal, Axial*	*Proton Density-Weighted and T1, T2-Weighted Imaging*	*Not specified*	*1.5 T and 3.0 T*
Gupta 2022	*Intelligent detection of knee injury in MRI exam*	*Random split: Training (80%),*	*Sagittal, Coronal, Axial*	*coronal T1 weighted, coronal T2 with fat saturation, sagittal proton density (PD)*	*Not specified*	*1.5 T and 3.0 T*

		Testing (20%)		*weighted, sagittal T2 with fat saturation, and axial PD weighted with fat saturation*		
Jeon 2021 (China)	*Interpretable and Lightweight 3-D Deep Learning Model for Automated ACL Diagnosis.*	*Random split: Training (80%), Validation (10%), Testing (10%)*	*Sagittal and Coronal*	*Proton Density-Weighted with Fat Suppression*	*3.0 mm*	*1.5 T and 3.0 T*
Jeon 2021 (MRNet)	*Interpretable and Lightweight 3-D Deep Learning Model for Automated ACL Diagnosis.*	*External validation only*	*Sagittal*	*coronal T1 weighted, coronal T2 with fat saturation, sagittal proton density (PD) weighted, sagittal T2 with fat saturation, and axial PD weighted with fat saturation*	*2.5–3.5 mm*	*1.5 T and 3.0 T*
Richardson 2021 (NFS)	*MR Protocol Optimization With Deep Learning: A Proof of Concept.*	*Random split: Training (80%), Validation (10%), Testing (10%)*	*Sagittal*	*PDW Fat-saturated (FS) and Non-fat-saturated (NFS) Imaging*	*Not specified*	*1.5 T and 3.0 T*
Germann 2020	*Deep Convolutional Neural Network-Based Diagnosis of Anterior Cruciate Ligament Tears: Performance Comparison of Homogenous Versus Heterogeneous Knee*	*Training (82.8%), Validation (8.6%), Testing (8.6%)*	*Sagittal, Coronal*	*coronal T1-weighted, coronal short tau inversion recovery (STIR), axial fat-suppressed intermediate-weighted*	*2.5–3 mm*	*1.5 T and 3.0 T*

	MRI Cohorts With Different Pulse Sequence Protocols and 1.5-T and 3-T Magnetic Field Strengths.			*(IW), and sagittal fat-suppressed and non–fat-suppressed IW sequences.*		
Liu 2019	*Fully Automated Diagnosis of Anterior Cruciate Ligament Tears on Knee MR Images by Using Deep Learning.*	*Random split: Training (57%), Validation (14%), Testing (29%)*	*Sagittal, Coronal, Axial*	*Proton Density-Weighted and Fat-Suppressed T2-Weighted Fast Spin Echo*	*2.0–3.0 mm*	*3.0 T (GE Healthcare Signa HDx)*
Astuto 2021	*Automatic Deep Learning-assisted Detection and Grading of Abnormalities in Knee MRI Studies.*	*Training (70%), Validation (15%), Testing (15%)*	*Sagittal, Coronal, Axial*	*Standard axial fat-suppressed T2-weighted fast spin-echo sequence, standard sagittal proton density–weighted and fat-suppressed T2-weighted fast spin-echo sequences, and standard coronal proton density–weighted and fat-suppressed proton density–weighted fast spin-echo sequences*	*0.5 mm*	*3.0 T (GE Discovery 750HD)*
Li 2023	*Automated diagnosis of anterior cruciate ligament via a weighted multi-view network.*	*Random split: Training (70%), Validation (10%), Testing (20%)*	*Sagittal, Coronal, Axial*	*T1-weighted, T2-weighted Imaging with Fat Suppression, PD weigthed series*	*3.5 mm*	*1.5 T and 3.0 T*

Table s6: MRI Inputs
The table summarizes studies on deep learning for knee MRI analysis, detailing validation methods (e.g., random split, cross-validation), imaging planes (sagittal, coronal, axial), scan sequences (PDWI, T2WI), scan thickness, MRI strength (1.5 T or 3.0 T), and contrast weighting (e.g., fat suppression). It provides a quick reference for methodologies and technical setups across studies. DL = Deep Learning; CNN = Convolutional Neural Network; PDWI = Proton Density-Weighted Imaging; T2WI = T2-Weighted Imaging; FS = Fat Suppression; MRNet = Stanford University Knee MRI Dataset; GAN = Generative Adversarial Network; ACL = Anterior Cruciate Ligament.

Author	*Title*	*Imaging modalities*	*Preprocessing*	*Radiomic features*	*Quantitative Feature Details*	*Feature selection*	*Region of Interest (ROI) creation*
Cheng 2024	*Application of machine learning-based multi-sequence MRI radiomics in diagnosing anterior cruciate ligament tears.*	*T1-weighted imaging (T1WI) and proton density-weighted imaging (PDWI) MRI sequences*	*Normalization to reduce parameter variability; Gaussian filtering for noise reduction*	*First-order (intensity), shape (geometry), second-order (GLCM), and higher-order features*	*2032 features initially extracted; reduced to 48 features (26 T1WI, 22 PDWI)*	*ICC > 0.75, t-tests ($p < 0.05$), Spearman correlation < 0.9, LASSO regression*	*Manual segmentation using ITK-SNAP focusing on intercondylar fossa*
Xue 2024	*Approaching expert-level accuracy for differentiating ACL tear types on MRI with deep learning.*	*Sagittal proton density-weighted spectral attenuated inversion recovery (PDW-SPAIR) MRI sequences*	*Normalization, standardization, and data augmentation (e.g., flipping, shifting, scaling); U-Net-based CNN for segmentation*	*21 features including 2D shape, GLCM, and other texture metrics*	*Features included 2D shape (20%)C, texture, and GLCM; statistically significant features reduced using random forest and cross-validation*	*Spearman correlation analysis (threshold: 0.9), $p < 0.05$ for univariate analysis, rank-based scoring*	*Automatic segmentation using U-Net CNN trained on manually annotated ACL masks*
Chen 2023	*A transfer learning approach for staging diagnosis of anterior cruciate ligament injury on a new modified MR dual*	*Modified dual precision positioning thin-slice oblique sagittal fat suppression proton density-weighted*	*Data augmentation (rotation, flipping, zoom); EfficientNet-B0 pre-trained model fine-tuned for ACL grading*	*Radiomics used as secondary enhancement via features extracted by convolutional layers (e.g., F1-*	*Grading performed with ROI and whole-image data; included performance metrics like sensitivity,*	*Transfer learning: pre-trained EfficientNet-B0 with dropout layers and classification refinement*	*Semi-automated segmentation: ROI extracted by program and evaluated for training/testing efficiency*

	precision positioning of thin-slice oblique sagittal FS-PDWI sequence.	*imaging (DPP-TSO-Sag-FS-PDWI)*		*score, Hinge Loss, J-score)*	*specificity, and accuracy per grade*		
Dung 2023	*End-to-end deep learning model for segmentation and severity staging of anterior cruciate ligament injuries from MRI.*	*Sagittal fat-saturated proton density (PD) fast spin-echo (FSE) MRI sequences*	*Normalization, resampling to 256×256 matrix; manual segmentation by expert radiologists; pseudo-masking for data augmentation*	*First-order features (e.g., minimum, mean, skewness), GLCM (e.g., correlation)*	*Radiomic features ranked by SelectKBest and statistical analysis (t-tests); P values significant for intact vs. fully ruptured ACL*	*SelectKBest for radiomic feature ranking; t-tests for statistical validation*	*Manual segmentation peer-reviewed by expert radiologists, followed by pseudo-labelling for unsegmented datasets*
Liang 2023	*Effective automatic detection of anterior cruciate ligament injury using convolutional neural network with two attention mechanism modules*	*Sagittal MRI T1-weighted sequences*	*Normalization to 320 × 320 pixels; data augmentation including flipping, rotation, scaling*	*Deep learning-based features using a CNN with ATM1 and ATM2*	*Accuracy: 80.63%; Precision: 77.41%; Sensitivity: 92.68%; Specificity: 65.09%; F1 Score: 84.36%; AUC: 88.86%*	*ATM1 (spatial attention for lesion localization); ATM2 (channel attention for classification enhancement)*	*Sagittal MRI sequences annotated by three orthopaedic surgeons; ACL labelled as injured or intact; validated by an expert*

Table s7- Radiomic Inputs
This table provides a comparative summary of studies employing radiomics and machine learning for diagnosing and staging anterior cruciate ligament (ACL) injuries using magnetic resonance imaging (MRI). It highlights the imaging modalities used (e.g., T1-weighted imaging [T1WI], proton density-weighted imaging [PDWI], proton density-weighted spectral attenuated inversion recovery [PDW-SPAIR], and dual precision positioning thin-slice oblique sagittal fat suppression proton density-weighted imaging

[DPP-TSO-Sag-FS-PDWI]), preprocessing techniques such as normalization, augmentation, and segmentation, and the types of radiomic features extracted, including first-order, texture (e.g., grey level co-occurrence matrix [GLCM]), and shape features. Quantitative details, such as the number of features analysed and diagnostic performance metrics, are listed alongside feature selection methods like interclass correlation coefficient (ICC), t-tests, and least absolute shrinkage and selection operator (LASSO). Region of interest (ROI) creation methods vary from manual segmentation by radiologists to automated or semi-supervised approaches, demonstrating a range of strategies for defining ROIs. This table serves as a resource for understanding key methodologies and outcomes in advancing ACL diagnostics using radiomics and convolutional neural networks (CNNs). ACL: Anterior Cruciate Ligament; MRI: Magnetic Resonance Imaging; T1WI: T1-Weighted Imaging; PDWI: Proton Density-Weighted Imaging; PDW-SPAIR: Proton Density-Weighted Spectral Attenuated Inversion Recovery; FSE: Fast Spin Echo; ROI: Region of Interest; GLCM: Gray Level Co-occurrence Matrix; ICC: Interclass Correlation Coefficient; LASSO: Least Absolute Shrinkage and Selection Operator; DPP-TSO-Sag-FS-PDWI: Dual Precision Positioning Thin-Slice Oblique Sagittal Fat Suppression Proton Density-Weighted Imaging; CNN: Convolutional Neural Network.

Study	*Automation*	*Interpretability Techniques*	*External Validation*
Wang M 2024	*Fully automated*	*-*	*Knee MRI, MRNet*
Awan 2021	*Manual pre-processing**	*-*	*KneeMRI*
Dung 2023	*Manual pre-processing**	*GLCM, SelectKBest*	
Mahmood 2024	*Manual pre-processing*	*-*	*-*
Chen 2023	*Semi-automatic**	*-*	*-*
Xue 2024	*Supervised Automated*	*-*	*-*
Namiri 2020	*Fully automated*	*-*	*-*
Wang J 2024	*Fully automated*	*T-SNE*	*-*
Wang X 2024	*Fully automated*	*-*	*-*
Wang D 2024	*Fully automated*	*-*	*-*
Wang 2024	*Fully automated*	*-*	*-*
Cheng 2024	*Manual pre-processing*	*LASSO*	*-*
Liang 2023	*Fully automated*	*Attention map*	*-*
Chen 2022	*Fully automated**	*-*	*-*
Awan 2023	*Fully automated*	*Attention map*	*-*
Joshi 2022	*Fully automated**	*-*	*-*
Shin 2022	*Fully automated**	*Grad-CAM*	*-*
Tran 2022	*Manual pre-processing**	*Heatmap*	*KneeMRI and MRNet*
Minamoto 2022	*Fully automated*	*-*	*-*
Sridhar 2022	*Manual pre-processing*	*-*	*-*
Zhang 2020	*Fully automated**	*-*	*-*
Chang 2019	*Fully automated*	*-*	*-*
Bien 2018	*Fully automated*	*-*	*MRNet*
Joshi 2023	*Fully automated*	*-*	*-*
Sun 2023	*Manual pre-processing*	*-*	*-*
Zhang 2024	*Manual pre-processing*	*-*	*-*
Sharma 2022	*Manual pre-processing*	*-*	*-*
Shakhovska 2022	*Manual pre-processing*	*-*	*MRNet*
Lin 2023	*Fully automated**	*Special attention graphs*	*-*

Gupta 2022	*Fully automated**	*-*	*-*
Jeon 2021	*Fully automated**	*-*	*Chiba, MRNet*
Richardson 2021	*Manual pre-processing**	*-*	*-*
Germann 2020	*Fully automated*	*-*	*-*
Liu 2019	*Fully automated*	*Probability map*	*-*
Astuto 2021	*Fully automated*	*Probability map*	*External dataset (Unspecified)*
Li 2023	*Fully automated**	*-*	*-*

Table s8 - Automation, explainability and external validation.
**= unclear*
